# An HR2-Mimicking Sulfonyl-γ-AApeptide Is a Potent Pan-coronavirus Fusion Inhibitor with Strong Blood–Brain Barrier Permeability, Long Half-Life, and Promising Oral Bioavailability

**DOI:** 10.1021/acscentsci.3c00313

**Published:** 2023-04-28

**Authors:** Songyi Xue, Wei Xu, Lei Wang, Xinling Wang, Qianyu Duan, Laurent Calcul, Shaohui Wang, Wenqi Liu, Xingmin Sun, Lu Lu, Shibo Jiang, Jianfeng Cai

**Affiliations:** † Department of Chemistry, University of South Florida, 4202 East Fowler Avenue, Tampa, Florida 33620, United States; ‡ Key Laboratory of Medical Molecular Virology (MOE/NHC/CAMS), School of Basic Medical Sciences, Shanghai Frontiers Science Center of Pathogenic Microbes and Infection, Shanghai Institute of Infectious Disease and Biosecurity, Fudan University, Shanghai 200433, China; § Department of Molecular Medicine, Morsani College of Medicine, University of South Florida, Tampa, Florida 33620, United States

## Abstract

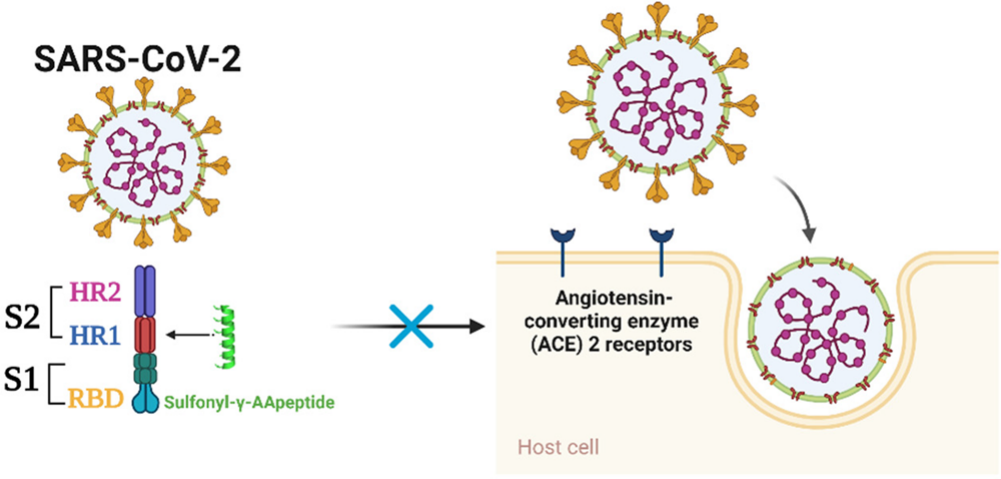

Neutralizing antibodies and fusion inhibitory peptides have the potential required to combat the global pandemic caused by SARS-CoV-2 and its variants. However, the lack of oral bioavailability and enzymatic susceptibility limited their application, necessitating the development of novel pan-CoV fusion inhibitors. Herein we report a series of helical peptidomimetics, d-sulfonyl-γ-AApeptides, which effectively mimic the key residues of heptad repeat 2 and interact with heptad repeat 1 in the SARS-CoV-2 S2 subunit, resulting in inhibiting SARS-CoV-2 spike protein-mediated fusion between virus and cell membranes. The leads also displayed broad-spectrum inhibitory activity against a panel of other human CoVs and showed strong potency *in vitro* and *in vivo*. Meanwhile, they also demonstrated complete resistance to proteolytic enzymes or human sera and exhibited extremely long half-life *in vivo* and highly promising oral bioavailability, delineating their potential as pan-CoV fusion inhibitors with the potential to combat SARS-CoV-2 and its variants.

## Abstract 2

HR2-mimicking sulfonyl-γ-AApeptides disrupted HR1/HR2 PPI to stop the entry of SARS-CoV-2 and inhibited the infection of SARS-CoV-2 and other emerging SARSr-CoVs both *in vitro* and *in vivo*.

## Introduction

COVID-19, caused by SARS-CoV-2, is associated with more than 663 million confirmed cases and 6.7 million deaths as of January 7, 2023.^1^ Although several vaccines^2^ and small-molecule drugs^3^ have now been authorized or approved for human use, the consistent emergence of new viral variants has quickly jeopardized their efficacy.^4^ Therefore, it is imperative to continue developing alternative and broad-spectrum prophylactics and therapeutics to combat this pandemic.

**Figure 1:**
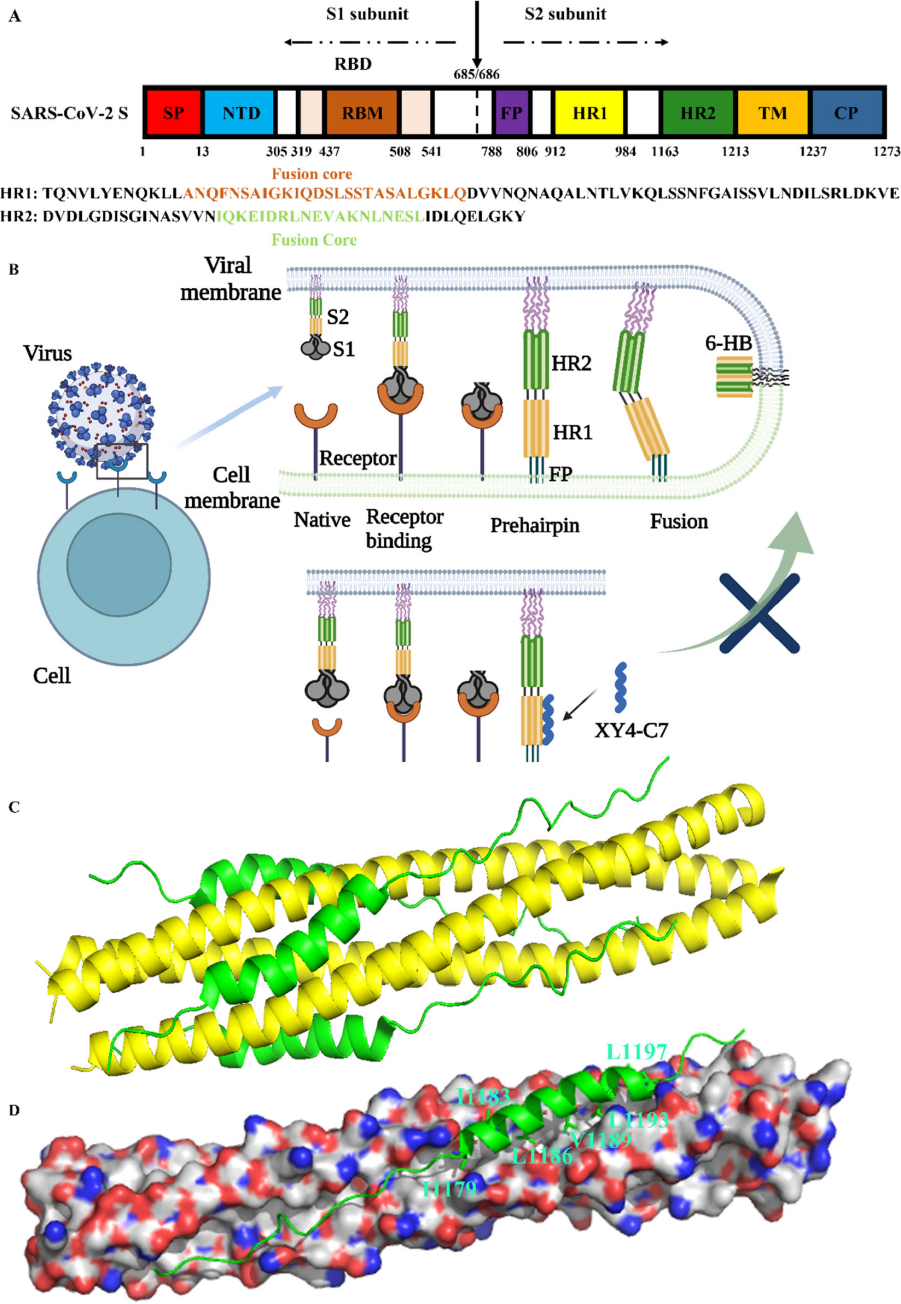
Fusion process of SARS-CoV-2 infection and proposed inhibitory mechanism of sulfonyl-γ-AApeptides. (A) Schematic representation of SARS-CoV-2 spike protein. SP, signal peptide; NTD, N-terminal domain; RBD, receptor-binding domain; RBM, receptor-binding motif; FP, fusion peptide; HR1, heptad repeat 1; HR2, heptad repeat 2; TM, transmembrane domain; CP, cytoplasm domain. (B) Fusion process mediated by S protein of SARS-CoV-2 and proposed mechanism of sulfonyl-γ-AApeptides to inhibit the infection of SARS-CoV-2. (C) Side view of the crystal structure of 6-HB formed by the association between HR2 and HR1 (PDB code 6LXT). (D) Binding interaction of key residues on HR2 (green) with the pockets on HR1 trimer.

SARS-CoV-2 is a member of the group of highly mutable β-coronaviruses, enabling them to adapt to new hosts and ecological niches.^5^ Its viral particles are composed of four structural proteins: spike (S), envelope (E), membrane (M), and nucleoprotein (N) proteins.^6^ These proteins play significant roles in the viral life cycle and are common to all human coronaviruses (HCoVs), making them prospective targets for the development of broad-spectrum antiviral agents.^7^ Among them, S protein facilitates viral entry into target cells by recruiting the cellular serine protease TMPRSS2 for S protein priming and the angiotensin-converting enzyme 2 (ACE2) as the entry receptor (Figure 1A).^8,9^ S protein has two functional subunit domains (Figure 1A,B), S1 and S2.^10^ The S1 subunit binds with the ACE2 receptor through its receptor-binding domain (RBD), followed by conformational changes in the S2 subunit that allow the fusion peptide domain (FP) to insert into the cell membrane of host target cells. The heptad repeat 1 (HR1) region in the S2 subunit assembles into a homotrimeric structure, exposing three highly conserved hydrophobic grooves that interact with heptad repeat 2 (HR2) (Figure 1C,D) to form a six-helical bundle (6-HB) structure that brings the viral and cellular membranes close together to start the viral fusion process (Figure 1B).^7,11^ The RBD and HR regions are excellent targets for the development of specific therapeutics aimed at the fusion process.^12^ Although RBD-based antibodies have been validated to effectively prevent virus attachment to ACE2,^13^ it is extremely challenging to design a broad-spectrum antiviral drug that targets the RBD because of its highly mutable nature.^14,15^ In contrast, it is generally agreed that HR1 could serve as a good target for the development of pan-CoV fusion inhibitors against highly pathogenic HCoVs since it is highly conserved among diverse HCoVs.^7,16^

A few peptide-based fusion inhibitors targeting HR1 have now been developed, and they have shown considerable effectiveness in preventing SARS-CoV-2 infection *in vitro* and *in vivo*.^7,16−21^ Jiang and colleagues identified two pan-CoV fusion inhibitors, EK1^7^ and EK1C4,^16^ which could significantly inhibit the fusion of diverse HCoVs, including SARS-CoV-2, SARS-CoV, MERS-CoV, HCoV-NL63, and HCoV-OC43. Zhu et al.^17^ created a lipopeptide fusion inhibitor, IPB02, which was highly effective in preventing SARS-CoV-2 S-protein-mediated cell–cell fusion and pseudovirus infection. Daily intranasal treatment of [SARS_HRC_-PEG_4_]_2_-chol, a dimeric lipopeptide fusion inhibitor developed by de Vries et al.,^18^ to SARS-CoV-2-infected ferrets could entirely stop SARS-CoV-2 direct-contact transmission. However, these bioactive peptides have intrinsically low biostability and bioavailability due to their canonical peptide backbone, which results in a short half-life and makes them challenging to use as oral medications.^22^

**Figure 2:**
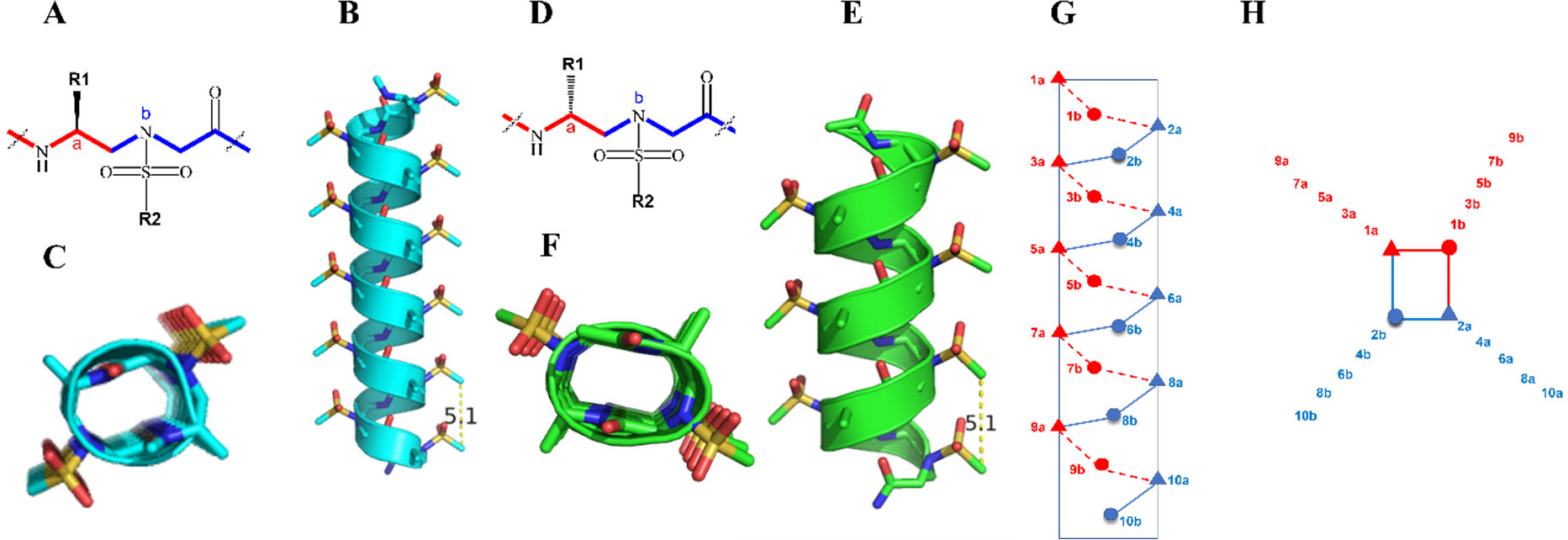
Chemical and crystal structure of sulfonyl-γ-AApeptides. (A) Chemical structure of l-sulfonyl-γ-AApeptides. **a** and **b** denote the chiral side chain and the sulfonamide side chain from the building block, respectively. (B) Crystal structure of l-sulfonyl-γ-AApeptide. (C) Top view of (B). (D) Chemical structure of d-sulfonyl-γ-AApeptides. **a** and **b** denote the chiral side chain and the sulfonamide side chain from the building block, respectively. (E) Crystal structure of d-sulfonyl-γ-AApeptide. (F) Top view of (E). (G, H) Schematic representation of the distribution of side chains from sulfonyl-γ-AApeptides shown from (G) side view and (H) top view of the helical wheel.

Non-natural sequence-specific peptidomimetics have become a promising alternative strategy to modulate protein–protein interactions (PPIs)^23−27^ to alleviate issues associated with the intrinsic drawbacks of peptides. In addition to retaining the advantages of natural peptides, foldameric peptidomimetics also exhibit unique structures and functions and are highly resistant to enzymatic hydrolysis.^23,28^ We have developed a new class of peptidomimetics, γ-AApeptides^28−30^ (oligomers of *N*-acyl-*N*-aminoethyl amino acids), based on the γ-chiral peptide nucleic acid (PNA) backbone. They show extraordinary resistance to proteolytic degradation and amenability to chemical diversification, making them suitable candidates for a variety of biological applications.^12,31−34^ As a subclass of γ-AApeptides, sulfonyl-γ-AApeptides (Figure 2A) not only possess the merits noted above but can also adopt well-defined helical structures^33−36^ (Figure 2B,C). Notably, the sulfonyl-γ-AApeptide helix displays a more robust and stable helical conformation than the α-helix of the same length, presumably from the intramolecular hydrogen bonding and the curved nature of sulfonamide moieties in the molecular framework. It is well-known that homogeneous l-sulfonyl-γ-AApeptides adopt left-handed 4_14_-helix helical conformations with four side chains per turn and helical pitch of 5.1 Å^35^ (Figure 2B,C). Since these are characteristics analogous to α-helix, albeit with different handedness, they have been designed to mimic the helical domain of proteins and have been shown to effectively modulate a number of PPIs, such as BCL9,^37^ p53,^38^ GLP-1,^39^ and VEGF.^40,41^ It should be noted that the left-handedness in the sequences could be switched to right-handedness by changing l-sulfonyl-γ-AA building blocks to d-sulfonyl-γ-AA building blocks (Figure 2D), leading to right-handed helices that are expected to further facilitate the design of mimetics of α-helix due to their closer similarity (Figure 2E,F).^36,42^ We envisioned that the molecular scaffold of d-sulfonyl-γ-AApeptide could be adopted, through rational design, to inhibit the viral fusion process of SARS-CoV-2.

To the best of our knowledge, there are no orally bioavailable fusion inhibitory peptides and no fusion inhibitors based on entirely unnatural foldameric scaffolds that can block the entry of SARS-CoV-2. Herein we report the design of right-handed helical d-sulfonyl-γ-AApeptides that mimic the hot spots of the HR2 peptide and disrupt the interaction between the HR1 and HR2 domains of the S2 subunit in SARS-CoV-2. By conjugating a cholesterol molecule to the lead sulfonyl-γ-AApeptides, we identified a liposulfonyl-γ-AApeptide (**XY4-C7**) which exhibited potent inhibitory activity against SARS-CoV-2 in the pseudovirus (PsV) and authentic virus infection assays, presenting a high selectivity index (SI). Additionally, **XY4-C7** demonstrated broad-spectrum antiviral activity against a range of coronaviruses, including SARS-CoV-2 and its Delta variant and other HCoVs like SARS-CoV, MERS-CoV, HCoV-NL63, and HCoV-OC43 as well as bat SARS-related coronavirus (SARSr-CoV) WIV1, which is consistent with the fact that both HR2 and HR1 domains are highly conserved across divergent coronaviruses. Moreover, administration of **XY4-C7** via the nasal route revealed highly prophylactic and excellent therapeutic effects in *in vivo* studies. Additionally, due to its unnatural backbone, **XY4-C7** showed remarkable resistance to proteolytic degradation and demonstrated a very long half-life and promising oral bioavailability in pharmacokinetic (PK) studies, suggesting that sulfonyl-γ-AApeptide has the potential to be developed into therapeutic and prophylactic drugs for the treatment and prevention of infection by SARS-CoV-2 and other HCoVs.

## Results

### Structural Insight of Six-Helical Bundle Formed between HR1 and HR2

The 6-HB formed by HR1 and HR2 domains is crucial to the membrane fusion mediated by SARS-CoV-2 S protein, and its crystal structure has recently been determined.^16^ As shown in Figure 1C,D, three HR1 molecules form a parallel trimeric coiled-coil center, which is surrounded by three antiparallel HR2 helices. Hydrophobic force drives the interaction between HR1 and HR2 domains, and this interaction is mainly located in the helical fusion core region. Each pair of two neighboring HR1 helices creates a substantial hydrophobic groove that serves as the binding site for hydrophobic residues such as I1179, I1183, L1186, V1189, L1193, and L1197 in the HR2 domain. All these hydrophobic residues are located on the same face of HR2 α-helix (Figure 1D). Additionally, side-chain-to-side-chain hydrophilic interactions also stabilize the bundle structure.

### Rational Design of Sulfonyl-γ-AApeptides to Mimic HR2 Peptide in the Fusion Core

**Figure 3:**
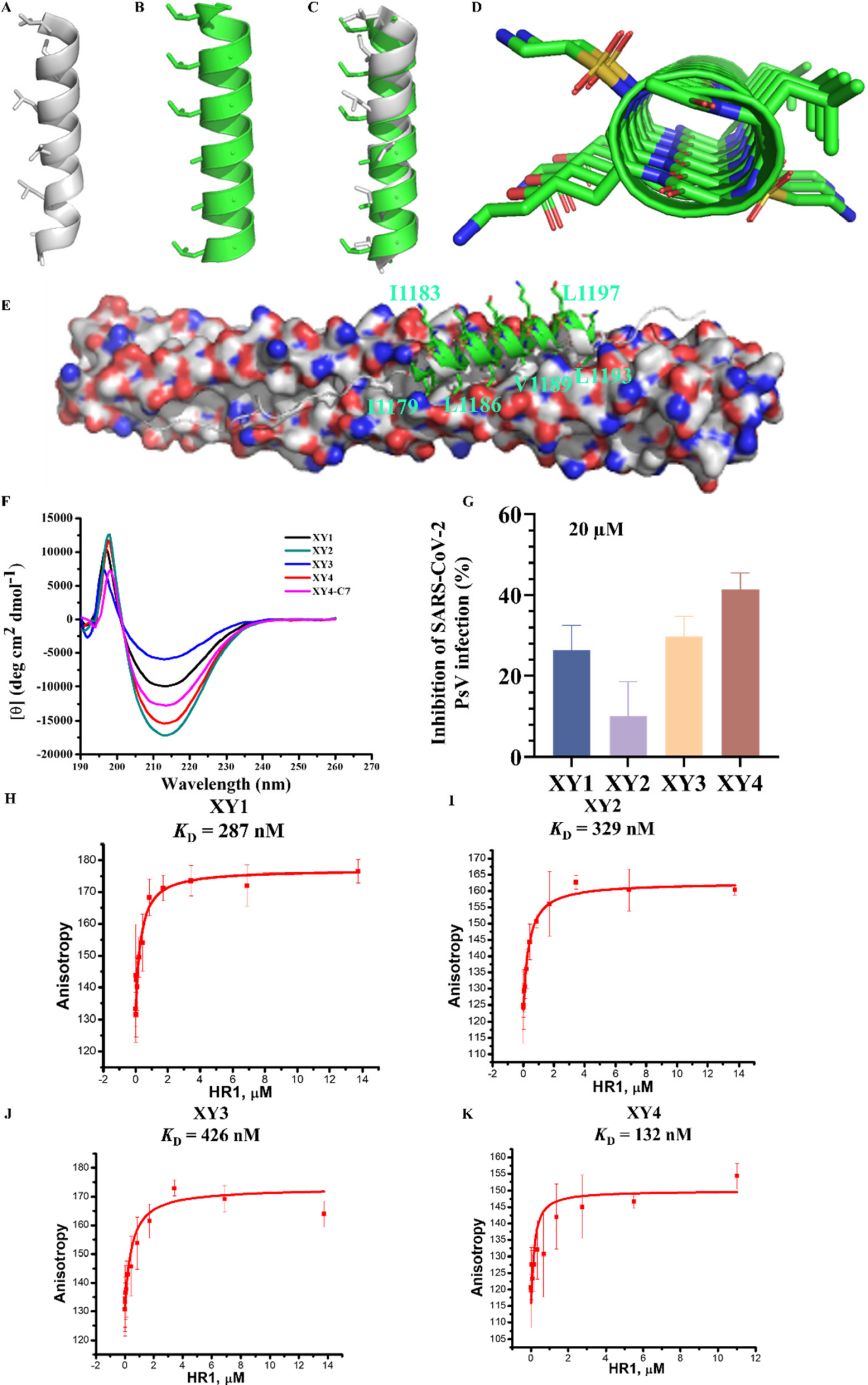
Rational design of sulfonyl-γ-AApeptide-based entry inhibitors and evaluation of their inhibitory activity *in vitro*. (A) Structure of HR2 peptide in fusion core (white). (B) Sulfonyl-γ-AApeptide mimic **XY4** (green). (C) Overlay of key binding residues between (A) and (B). (D) Top view of (B) with the polar side chains and key hydrophobic residues. (E) Superimposition of **XY4** (green) with critical residues of HR2 peptide in fusion core (white) on the binding surface of HR1. (F) CD spectra of sulfonyl-γ-AApeptides **XY1**–**XY4** and **XY4-C7** measured at 100 μM and room temperature in PBS buffer. (G) Inhibitory activity of four lead compounds (**XY1**, **XY2**, **XY3**, and **XY4**) at the concentration of 20 μM from SARS-CoV-2 pseudovirus infection assay. (H–K) Binding affinity between HR1 peptide and (H) **XY1**, (I), **XY2**, (J), **XY3**, and, (K) **XY4** determined by fluorescence polarization.

Based on the above analysis, we introduced six chiral hydrophobic residues at the 1a, 3a, 5a, 7a, 9a, and 11a positions on the same face of d-sulfonyl-γ-AApeptide helices to mimic the binding interaction of I1179, I1183, L1186, V1189, L1193, and L1197 of HR2, respectively, to reproduce binding affinity with HR1 (Figure 2D,H). Negatively and positively charged side chains were introduced to the sequences to form the salt bridges in the other two faces to enhance the helical stability and solubility of the sequences (Table 1). We first designed and synthesized four sequences (**XY1**–**XY4**, Table 1) bearing different sizes of hydrophobic groups at positions 1b, 8b, and 10b, as these residues reside in the hydrophobic groove of HR1. We first performed fluorescence polarization assays to evaluate the binding affinity of these sequences toward the HR1 peptide (911–987). As expected, all four sequences showed an excellent binding affinity with the HR1 peptide with *K*_d_ values from 0.13 to 0.42 μM (Table 1). This is consistent with the modeling, in which the crucial residues of HR2 (Figure 3A) and the hydrophobic side chains of **XY4** (Figure 3B) overlap very well (Figure 3C). The overlay of **XY4** with the HR2 peptide on the surface of HR1 (Figure 3E) also suggested that **XY4** could recognize the hydrophobic cleft of HR1 effectively. Indeed, all d-sulfonyl-γ-AApeptides assumed typical right-handed helical structures in solution. As shown in Figure 3F, CD experiments were carried out and revealed strong negative Cotton effects between 205 and 215 nm,^42^ which is a mirror image of the CD signature of left-handed l-sulfonyl-γ-AApeptides, implying that these d-sulfonyl-γ-AApeptides adopted right-handed helical conformations, which is similar to α-helical peptides.

**Table 1:**
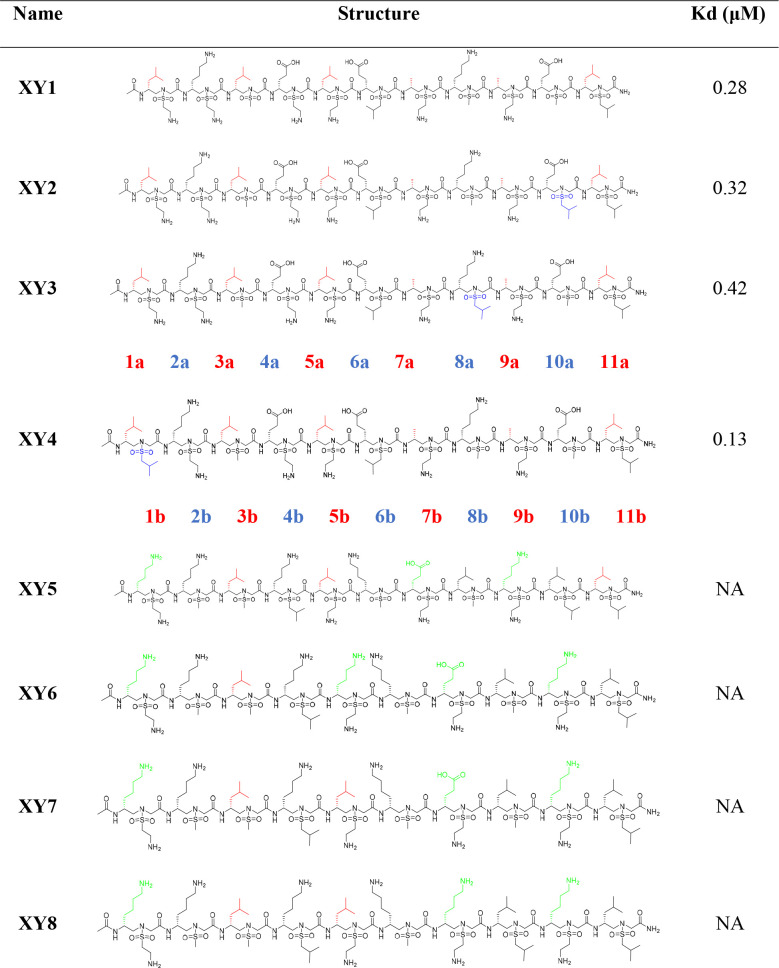
Structures of Selected Sulfonyl-γ-AApeptide Helical Mimics (**XY1**–**XY8**)^a^

a Critical binding residues are shown in red. The binding affinities (*K*_d_) of sequences to the HR1 peptide were determined by fluorescence polarization. The amino acid sequence of the HR2 peptide in the fusion core is SVVNIQKEIDRLNEVAKNLNESLIDLQ.

Next, we tested the inhibitory activity of these four sequences at the concentration of 20 μM against SARS-CoV-2 infection *in vitro* using our well-established SARS-CoV-2 PsV infection assay with Caco-2 cells. While not highly potent, all of them exhibited a certain level of inhibitory activity, particularly compound **XY4**, which inhibited PsV infection by roughly 40% at this concentration (Figure 3G). We also synthesized **XY5**, **XY6**, **XY7**, and **XY8** in which certain hydrophobic residues at positions 1a, 3a, 5a, 7a, 9a, and 11a were changed to hydrophilic groups (Table 1). As expected, their binding affinity with the HR1 peptide was very low, confirming that potent binding affinity results from successful mimicry of the critical hydrophobic residues in the HR2 peptide in the fusion core by sulfonyl-γ-AApeptides. As **XY4** displayed the most effective inhibitory activity, it was selected as our lead compound for further modification.

### **XY4-C7**, Sulfonyl-γ-AApeptides-PEG*n*-Chol, Demonstrated Excellent Fusion Inhibitory Activity and Moderate Cytotoxicity

**Figure 4:**
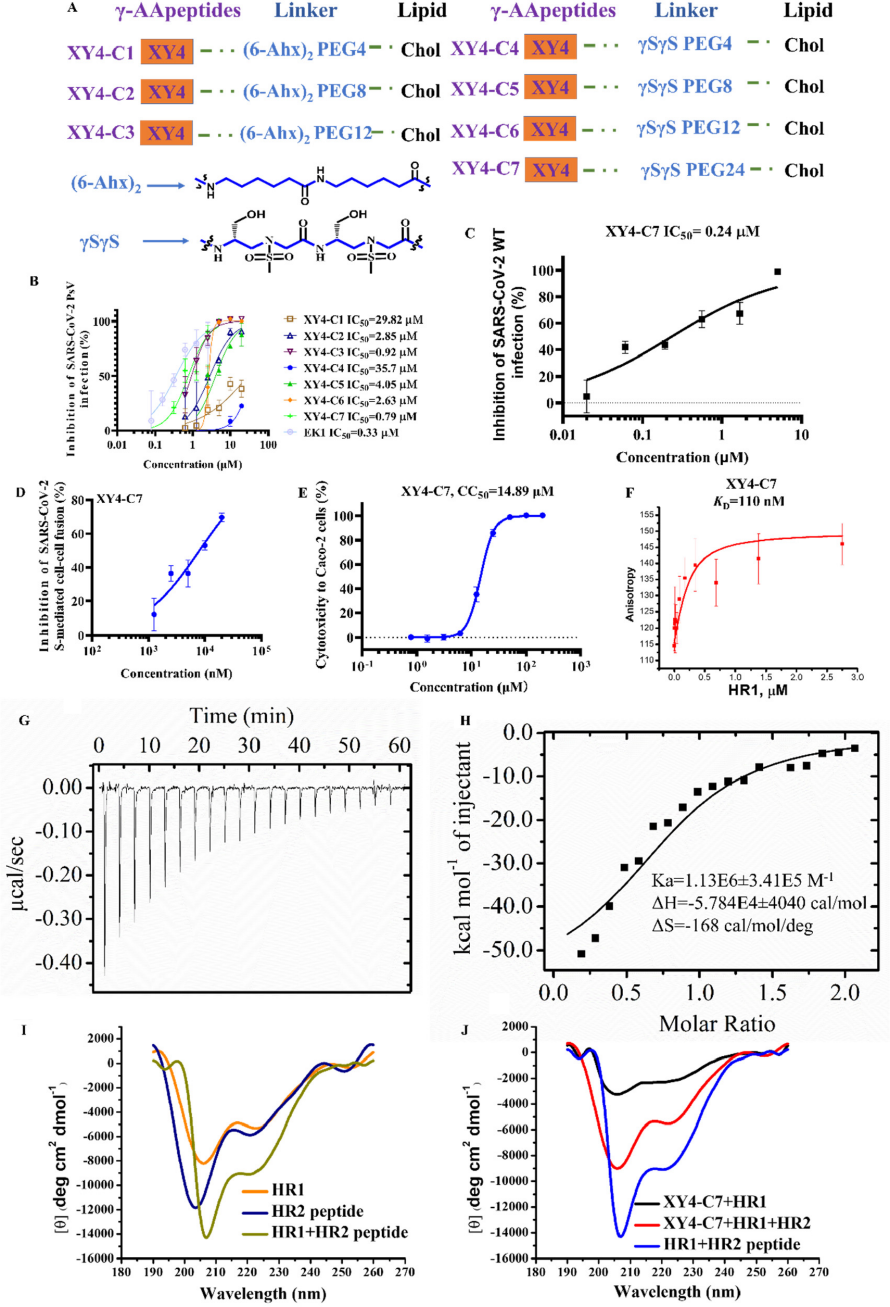
Rational design of sulfonyl-γ-AApeptides-PEG*n*-Chol inhibitors and evaluation of their antiviral activity *in vitro*. (A) Design diagram of sulfonyl-γ-AApeptides-PEG*n*-Chol, including **XY4-C1** to **XY4-C7**. (B) Inhibitory activity of sulfonyl-γ-AApeptides-PEG*n*-Chol on SARS-CoV-2 PsV infection. (C) Inhibitory activity of **XY4-C7** on authentic SARS-CoV-2 infection in Caco-2 cells. (D) Inhibitory activity of **XY4-C7** on SARS-CoV-2 S-mediated cell–cell fusion. (E) Cytotoxicity of **XY4-C7** to Caco-2 cells. (F) Affinity of binding between **XY4-C7** and the HR1 peptide determined by fluorescence polarization. (G, H) Affinity of binding between **XY4-C7** and the HR1 peptide determined by isothermal titration calorimetry (ITC). (I) CD spectra of SARS-CoV-2 HR1 peptide alone (orange), SARS-CoV-2 HR2 peptide (navy), and SARS-CoV-2 HR1/HR2 peptide complex (dark yellow). (J) CD spectra of **XY4-C7**/HR1 peptide mixture (black), **XY4-C7**/HR1 peptide/HR2 peptide mixture (red), and SARS-CoV-2 HR1/HR2 peptide complex (blue).

**XY4** could inhibit SARS-CoV-2 infection; however, its activity is considerably weaker than that of the recently reported pan-CoV fusion inhibitors EK1.^7^ Lipidation is a demonstrated strategy to enhance the antiviral activities of fusion inhibitors such as EK1C4^16^ by increasing their local concentration at the host cell’s membrane surface.^16,43^ Therefore, cholesterol (Chol) was covalently attached to the C-terminus of **XY4** with the assistance of different spacers, and the corresponding XY4-PEG*n*-Chols were constructed (Figure 4A). These XY4-PEG*n*-Chols were evaluated by SARS-CoV-2 PsV infection assay as indicated by half-maximal inhibitory concentration (IC_50_). First, we added two flexible linkers, Fmoc-6-aminohexanoic acid (6-Ahx) and Chol-poly(ethylene glycol) (PEG), with varied lengths of 4, 8, and 12 units. The IC_50_ values of these three compounds were determined to be 29.82, 2.86, and 0.93 μM, respectively (Figure 4B), indicating that lipidation modification was successful and that more units of PEG added would increase the inhibitory activity. We changed the 6-Ahx flexible linker to γSγS (Figure 4A) to obtain the other four sequences **XY4-C4**, **XY4-C5**, **XY4-C6**, and **XY4-C7**, which were inspired by the rigid linker (GSGSG) utilized by EK1C4.^16^ The anti-PsV activities of these four compounds were examined in Caco-2 cells, and their IC_50_ values were found to be 35.77, 4.05, 2.63, and 0.79 μM, respectively (Figure 4B), among which **XY4-C7** appeared to be much more potent than **XY4**, exhibiting even a better activity than the previously reported EK1 peptide (SLDQINVTFLDLEYEMKKLEEAIKKLEESYIDLKEL, IC_50_ = 2.38 μM).^7,16^ As a result, we chose **XY4-C7** as the lead compound to do further assessments. It is worth noting that **XY4-C7** effectively prevented authentic SARS-CoV-2 infection at the cellular level in a dose-dependent manner with an IC_50_ of 0.24 μM in Caco-2 cells (Figure 4C), consistent with the results from the PsV infection assay. The same cell line was used to evaluate its cytotoxicity, and the half-maximal cytotoxic concentration (CC_50_) was 14.89 μM (Figure 4E). The selectivity index (SI = CC_50_/IC_50_) was 62.04, suggesting that **XY4-C7** specifically inhibits SARS-CoV-2 entry into the host cells. The subsequent experiment also demonstrated that **XY4-C7** targeted the S protein and inhibited the SARS-CoV-2 S-protein-mediated cell–cell fusion in a dose-dependent manner (Figure 4D). The binding affinity of **XY4-C7** toward the HR1 peptide was determined by both fluorescence polarization (FP) assay and isothermal titration calorimetry (ITC) assay with IC_50_ values of 0.1 and 0.8 μM, respectively, suggesting that adding Chol to **XY4** did not significantly change the binding activity to the HR1 peptide (Figure 4F–H). After that, we employed circular dichroism (CD) spectoscopy to probe the mechanism of the inhibitory activity of **XY4-C7**. Both HR1 peptide and HR2 peptide exhibited the typical α-helicity in the solution; the mixture of HR1 peptide and HR2 peptide showed a more pronounced α-helical character (Figure 4I), which may indicate the formation of the HR1/HR2 complex. However, in the presence of **XY4-C7**, the intensity of the CD signature of the HR1 peptide decreased dramatically, implying a significant conformational change due to the interaction between the HR1 peptide and **XY4-C7**. In addition, the characteristic of α-helicity of 6-HB significantly decreased when **XY4-C7** was mixed with HR1 peptide/HR2 peptide together (Figure 4J), suggesting that **XY4-C7** could disrupt the formation of HR1/HR2 complex by potently binding with the HR1 (Figure 4F–H,J). Taken together, these results show that **XY4-C7** is a potent and selective inhibitor of SARS-CoV-2 infection with a high binding affinity toward the HR1 peptide to disrupt the formation of 6-HB between HR1 and HR2 fusion core.

### **XY4-C7** Efficiently Inhibited Infection by Authentic SARS-CoV-2 Delta Variant, Three Pseudotyped HCoVs, and One Pseudotyped Bat SARSr-CoV

**Figure 5:**
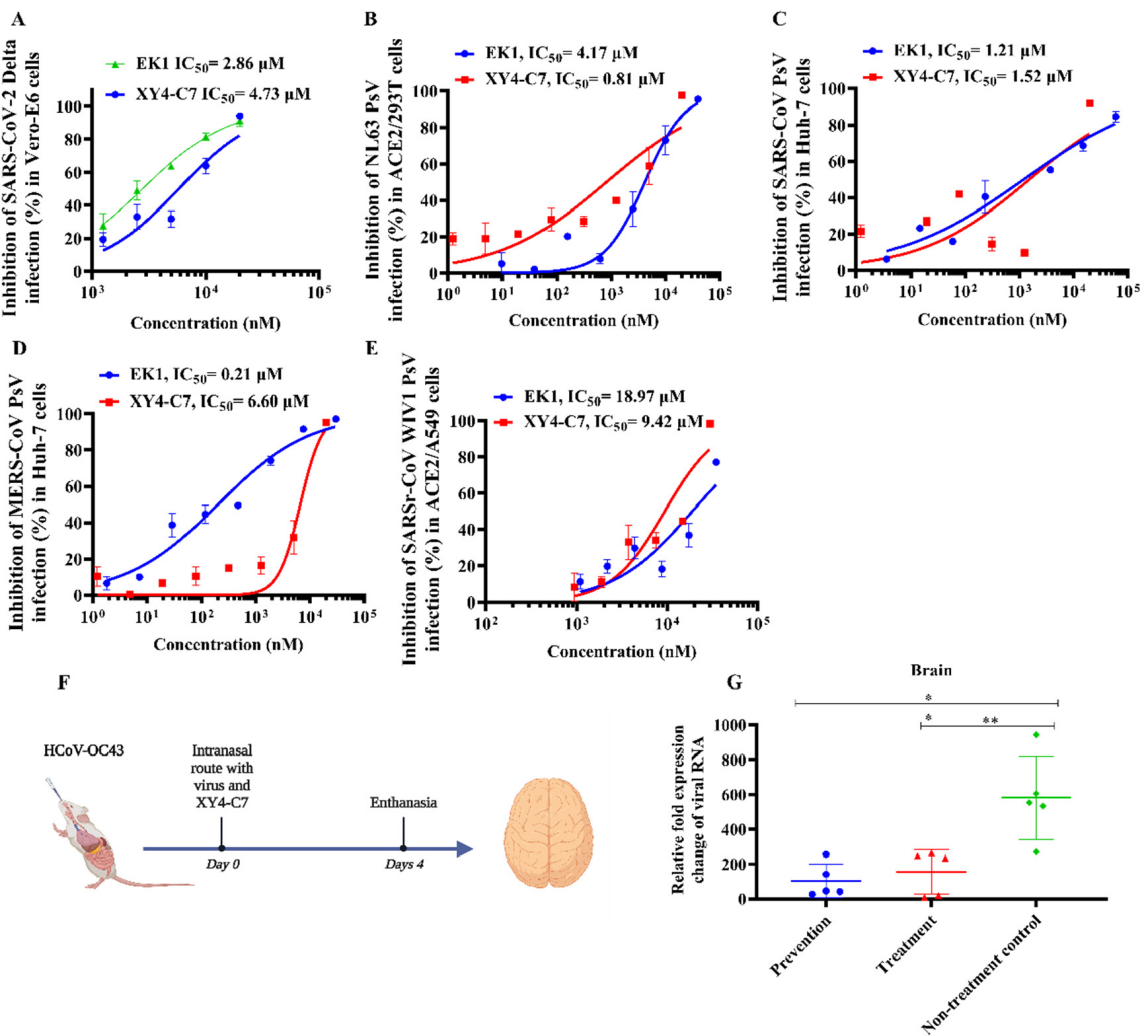
Inhibition of **XY4-C7** against infection by authentic SARS-CoV-2 Delta variant, three pseudotyped HCoVs, and one bat SARSr-CoV *in vitro* as well as HCoV-OC43 infection *in vivo*. (A) Inhibitory activity of **XY4-C7** against infection of authentic SARS-CoV-2 Delta variant in Vero-E6 cells. (B) Inhibitory activity of **XY4-C7** against pseudotyped HCoV-NL63 in ACE2/293T cells. (C) Inhibitory activity of **XY4-C7** against pseudotyped SARS-CoV in Huh-7 cells. (D) Inhibitory activity of **XY4-C7** against pseudotyped MERS-CoV in Huh-7 cells. (E) Inhibitory activity of **XY4-C7** against pseudotyped bat SARSr-CoV W1V1 in ACE2/A549 cells. (F) Schematic diagram of **XY4-C7** administration and HCoV-OC43 challenge. (G) *In vivo* efficacy of **XY4-C7** (1 mg/kg) against HCoV-OC43 infection in newborn mice. Viral RNA expression level in the brain tissue of mice in each group on the fourth day postinfection was detected.

To determine the breadth of **XY4-C7**, we tested its inhibitory activity against the SARS-CoV-2 Delta variant, three other HCoVs, and one bat SARSr-CoV. We found that **XY4-C7** is effective against authentic SARS-CoV-2 Delta variant infection in Vero-E6 cells with an IC_50_ value of 4.73 μM (Figure 5A). **XY4-C7** could also potently inhibit infection of pseudotyped SARS-CoV, MERS-CoV, and HCoV-NL63 as well as bat SARSr-CoV WIV1 in different cell lines with IC_50_ values ranging from 0.81 to 9.42 μM, confirming that **XY4-C7** is a pan-HCoV fusion inhibitor (Figure 5B–E). Overall, **XY4-C7** is a promising broad-spectrum antiviral agent that is effective against SARS-CoV-2 and Delta variant as well as other HCoVs and bat SARSr-CoV that may cause future coronavirus diseases.

### Intranasally Applied **XY4-C7** Potently Protected Newborn Mice against HCoV-OC43 Infection

We next employed a mouse model of HCoV-OC43 infection to investigate the protective efficacy of **XY4-C7** in clinical applications. **XY4-C7** was administered to OC43-infected newborn mice in prevention (*n* = 5) or treatment (*n* = 5) groups via the intranasal route at a low single dose of 1 mg/kg 0.5 h before or after challenge with HCoV-OC43 at 100 TCID_50_, respectively. Mice were sacrificed after 4 days, and brains were excised to determine viral load. As shown in Figure 5G, both prevention and treatment groups revealed significantly lower HCoV-OC43 RNA levels than the nontreatment group. This result suggests that **XY4-C7** can effectively protect newborn mice from infection of HCoV-OC43, and based on this evidence, it is plausible to anticipate that **XY4-C7** could effectively inhibit SARS-CoV-2 infection as well as infection by other HCoVs *in vivo*.

### **XY4-C7** Was Highly Stable in the Presence of Pronase or Human Sera

**Figure 6:**
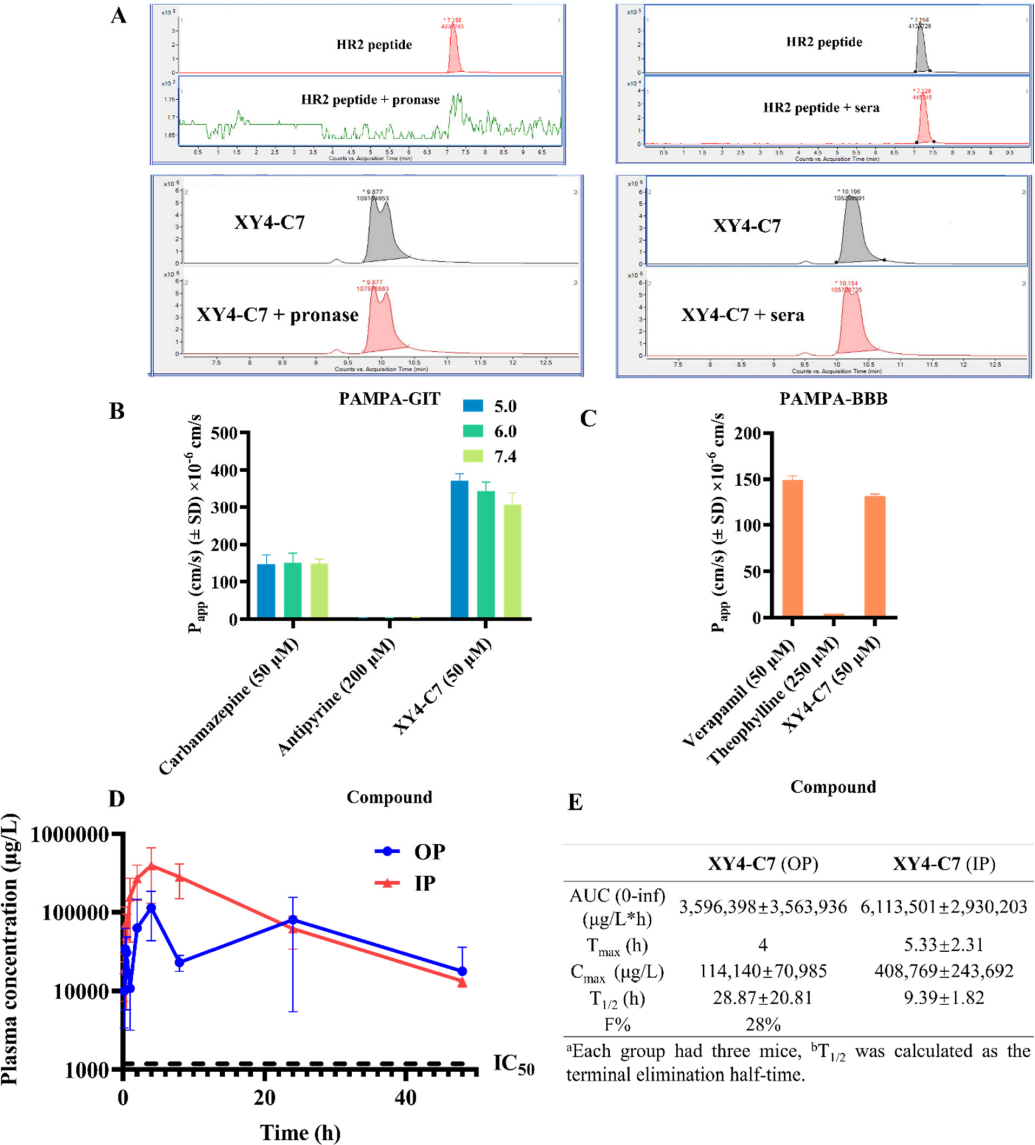
Evaluation of stability, membrane passive permeability, oral bioavailability, and PK profile of **XY4-C7** in a mouse model. (A) Stability studies of HR2 peptide mimics. LC/MS/MS of the indicated control and **XY4-C7** incubated in the presence of Pronase or human sera, respectively, for 24 h. (B) PAMPA-GIT for standards and **XY4-C7** at different pH conditions. (C) PAMPA-BBB for standards and **XY4-C7** at pH 7.4. (D) Time–concentration plot of **XY4-C7** in PK study. Plasma concentration and time curve following intraperitoneal (IP) administration (red) and oral (OP) administration (blue) of 30 mg/kg **XY4-C7** in C57BL/6 mice (data are presented as mean ± SD, *n* = 3). The dashed line shows the inhibition rate of 50%. (E) Pharmacokinetics parameters of **XY4-C7** over 48 h in mice.

The inherent susceptibility to degradation toward proteolytic enzymes is a major bottleneck for canonical peptides for the development of antiviral agents. To this end, we evaluated the stability of **XY4-C7** and the HR2 peptide in the presence of Pronase, a mixture of hydrolytic enzymes theoretically degrading peptides into single amino acids or human sera. The samples were incubated with Pronase or human sera for 24 h and analyzed with LC/MS/MS (Figure 6A). **XY4-C7** was highly stable and did not show noticeable degradation in 24 h; however, the HR2 peptide was completely degraded in Pronase and around 90% degraded in human sera.

### **XY4-C7** Demonstrated Favorable Passive Permeability to the Blood–Brain Barrier and Gastrointestinal Tract Membranes

The parallel artificial membrane permeability assay (PAMPA) is a type of high-throughput permeability assay that has been widely used in the pharmaceutical industry to assess drug candidates.^44^ To this end, we assessed **XY4-C7** with PAMPA for blood–brain barrier (BBB) permeability (PAMPA-BBB) and the PAMPA for gastrointestinal tract (GIT) permeability (PAMPA-GIT). Expected *P*_app_ values for favorable, medium, and low permeability are >20 × 10^–6^ cm/s, 1–20 × 10^–6^ cm/s, and <1 × 10^–6^ cm/s, respectively, in both assays. Since human intraluminal pH varies in the stomach, duodenum, ileum, cecum, and rectum, we needed to evaluate the permeability capacity of **XY4-C7** under various pH conditions for the PAMPA-GIT. We used the fully orally bioavailable drug carbamazepine (50 μM) as the positive control, which had higher *P*_app_ values of around 150 × 10^–6^ cm/s at three different pH values, and the poorly orally bioavailable drug antipyrine (200 μM) as the negative control, which had lower *P*_app_ values of <6 × 10^–6^ cm/s at the same pH values (Figure 6B). As shown in Figure 6B, **XY4-C7** (50 μM) displayed favorable permeability with much higher *P*_app_ values of 372 × 10^–6^, 344 × 10^–6^, and 306 × 10^–6^ cm/s at pH 5.0, 6.2, and 7.4, respectively. As a result, **XY4-C7** is expected to have very promising oral bioavailability *in vivo*.

Based on its potent ability to invade the central nervous system (CNS) and affect the function of particular nuclei or neural circuits, SARS-CoV-2 causes a variety of severe neurological symptoms and complications, including acute stroke, hyposmia, Guillain-Barrè syndrome, and encephalitis.^45^ As such, we employed the PAMPA-BBB assay to evaluate the potential of **XY4-C7** to cross the BBB and predict its ability to prevent SARS-CoV-2 from invading the CNS, thus controlling these symptoms and complications. Verapamil, the positive control, could easily cross the BBB with a *P*_app_ value of 148 × 10^–6^ cm/s at a concentration of 50 μM (Figure 6C). In contrast, theophylline, the negative control, had a low *P*_app_ value of 4 × 10^–6^ cm/s, even at a concentration of 250 μM, and could barely cross the BBB (Figure 6C). Like verapamil, **XY4-C7** (50 μM) easily passed through the BBB with a *P*_app_ value of 132 × 10^–6^ cm/s, which may help to explain why it demonstrated potent protection of mouse brain against HCoV-OC43 infection in the HCoV-OC43-infected mouse model (Figure 6C). Therefore, we can predict that **XY4-C7** will have very promising potential to control SARS-CoV-2 in the CNS.

### **XY4-C7** Has a Favorable Pharmacokinetic Profile with Much Longer Half-Life and Very Promising Oral Bioavailability in Mouse

To determine the oral absorption and *in vivo* stability of **XY4-C7**, we performed PK studies in mice via intraperitoneal (IP) and oral (OP) administration of **XY4-C7** at 30 mg/kg over 48 h. In IP administration, the average maximum blood concentration (*C*_max_) of 408,769 μg/L was achieved within 5 h, while in OP administration, *C*_max_ of 114,140 μg/L was reached after 4 h (Figure 6D,E). This suggests that effective blood exposure was approximately 350-fold and 97-fold higher than the IC_50_ of **XY4-C7** in IP and OP administration, respectively. Additionally, the effective period of both administrations is over 24 h, and the plasma concentration decayed with a longer half-life (*t*_1/2_) of 9.4 h in IP administration and a significantly longer *t*_1/2_ of 28.9 h in OP administration (Figure 6D,E). Most importantly, **XY4-C7** again displayed high oral bioavailability (*F*) of 28%, indicating that it possesses the promising potential for use as an orally delivered drug (Figure 6D,E).

## Discussion

To combat the SARS-CoV-2 pandemic as well as emerging and re-emerging HCoVs in the future, particularly among the unvaccinated segment of the global population and rising concerns about drug resistance to various variants, it is urgently necessary to develop long-acting oral drugs with broad-spectrum activity across HCoVs. Bioactive peptides like EK1, EK1C4, and [SARS_HRC_-PEG_4_]_2_-chol, which were designed as pan-CoV fusion inhibitors, have already shown potent inhibitory activity against SARS-CoV-2.^7,16,18^ However, their use as long-acting oral drugs is challenging because of their limited bioavailability and biostability by the lack of a native peptide backbone. Peptidomimetics, which are designed to mimic the structure and function of bioactive peptides and proteins, have shown remarkable applications in protein surface mimicry and recognition, modulation of PPIs, and catalysis. Recently, we created and applied sulfonyl-γ-AApeptides as a new helical framework to design protein helical domain mimetics and modulate a variety of medicinally relevant PPIs, including VEGF/VEGFR, p53/MDM2, GLP-1, BCL9/β-catenin, and others. Most of these were constructed from l-sulfonyl-AApeptide building blocks, which have left-handed helical conformations, in contrast to the right-handedness of α-peptides. In this article, we have reported d-sulfonyl-γ-AApeptide-based right-handed helical foldamers, which were anticipated to display right-handed helical conformation more in line with that of α-helix, to mimic the HR2 peptide in the fusion core and thus prevent the SARS-CoV-2 fusion process.

The current design was based on the crystal structure of the HR2 fusion core in a complex with HR1 trimer. Most critical residues of the HR2 helix in the fusion core, including Ile1179, Ile1183, Leu1186, Val1189, Leu1193, and Leu1197, are involved in binding HR1 trimer. Therefore, some sulfonyl-γ-AApeptides were designed based on the helical structures to reproduce these hydrophobic functionalities using the chiral side chains at 1a, 3a, 5a, 7a, 9a, and 11a, respectively, on the same face of sulfonyl-γ-AApeptide foldamers. Hydrophilic groups were included on the other two faces of our foldamers, which are not involved in the interaction with the HR1 region in the fusion core, to improve the helical stability and solubility. Based on these design principles, we have shown that some sulfonyl-γ-AApeptides exhibit excellent binding affinity and strong interaction with the hydrophobic surface of the HR1 peptide. These results demonstrated that these sulfonyl-γ-AApeptides successfully mimicked the HR2 peptide in the fusion core by interacting with the HR1 trimer. Following validation by PsV infection assay, the lead sequence **XY4** was chosen for further optimization. Since lipidation is known to improve the efficacy of fusion inhibitors, we added two different linkers, the rigid and the flexible one, along with various PEG lengths with Chol. We found that **XY4-C7** retained its binding affinity and interacting capability with the HR1 peptide and exhibited highly potent activity in the authentic SARS-CoV-2 infection assay with IC_50_ of 0.24 μM and SI of 62. Moreover, **XY4-C7** is also highly effective against infection by authentic SARS-CoV-2 Delta variant and the pseudotyped SARS-CoV, MERS-CoV, and HCoV-NL63 as well as SARSr-CoV WIV1 from the bat. Following the *in vitro* test, we found that intranasally applied **XY4-C7** to newborn HCoV-OC43 mice potently inhibited its infection *in vivo*. Most importantly, *in vitro* and *in vivo* PK studies further proved that **XY4-C7** was highly resistant to proteolytic degradation and had an extremely long half-life and very promising oral bioavailability.

## Conclusion

We have identified several unnatural helical foldameric mimetics of the HR2 peptide in the fusion core in the S2 subunit of the SARS-CoV-2 S protein. Upon validation, we have found that the lead compound, **XY4-C7**, is a highly potent pan-CoV fusion inhibitor against infection by SARS-CoV-2 and its Delta variant and several HCoVs, including SARS-CoV, MERS-CoV, HCoV-NL63, and HCoV-OC43, as well as bat SARSr-CoV WIV1. Additionally, it showed outstanding PK properties in both PAMPAs and PK tests (both oral and intraperitoneal administrations). Therefore, it is reasonable to assume that **XY4-C7** can be further developed as a novel orally applicable anti-HCoV drug and that combining **XY4-C7** with other available COVID-19 therapeutics with different mechanisms of action may have a synergistic antiviral effect, resulting in a new cocktail for the treatment of infection of SARS-CoV-2 and other HCoVs. Overall, we believe that this work can be broadened to develop different antiviral agents using sulfonyl-γ-AApeptides as well as utilized to modulate thousands of other PPIs.

## Supporting Information Available

Detailed experimental conditions and methods, synthesis, characterization, and biological assays (PDF)
