# Supplementary material for: An HR2-Mimicking Sulfonyl-γ-AApeptide Is a Potent Pan-coronavirus Fusion Inhibitor with Strong Blood–Brain Barrier Permeability, Long Half-Life, and Promising Oral Bioavailability
Source: ACS Cent Sci. 2023 Apr 28;9(5):1046–58. doi: 10.1021/acscentsci.3c00313 (PMC10184535; doi:10.1021/acscentsci.3c00313)
Supplement: Supplementary file 1 — oc3c00313_si_001.pdf [file oc3c00313_si_001.pdf]

An HR2-mimicking sulfonyl-γ-AApeptide is a potent pan-coronavirus fusion inhibitor with strong blood-brain barrier permeability, long half-life and promising oral bioavailability

Songyi Xue,[1,+] Wei Xu,[2,+,*] Lei Wang,[1] Xinling Wang,[2] Qianyu Duan,[2] Laurent Calcul,[1] Shaohui Wang,[3] Wenqi Liu,[1] Xingmin Sun,[3] Lu Lu,[2,*] Shibo Jiang[2,*], and Jianfeng Cai[1,*]

[1]Department of Chemistry, University of South Florida, 4202 E. Fowler Ave., Tampa, FL, United States.

[2]Key Laboratory of Medical Molecular Virology (MOE/NHC/CAMS), School of Basic Medical Sciences, Shanghai Frontiers Science Center of Pathogenic Microbes and Infection, Shanghai Institute of Infectious Disease and Biosecurity, Fudan University, Shanghai, China.

[3]Department of Molecular Medicine, Morsani College of Medicine, University of South Florida, Tampa, FL, United States.

+ These authors contributed to the work equally.

Email: jianfengcai@usf.edu, shibojiang@fudan.edu.cn, lul@fudan.edu.cn and xuwei11@fudan.edu.cn

Table of Contents

1. Synthesis of sulfonyl-γ-AApeptide building blocks S3

1.1 General Information S3

1.2 Synthesis S3

1.3 Characterization of sulfonyl-γ-AApeptide building blocks S4

2. Synthesis of Chol-PEGn-bromoacetate S6

3. Preparation of sulfonyl-γ-AA peptide sequences mimicking the HR2 peptide on fusion core S8

3.1 General Information S8

3.2 Synthesis S8

3.2.1 Synthesis of sulfonyl-γ-AApeptides S8

3.2.2 Synthesis of Chol-PEGn-sulfonyl-γ-AApeptides S9

3.3 Characterization of sulfonyl-γ-AApeptides and Chol-PEGn-sulfonyl-γ-AApeptides S10

4. Fluorescence polarization assay S24

5. Circular Dichroism S24

6. PAMPA-BBB assay S25

7. PAMPA-GIT assay S26

8. ITC assay S27

9. Enzyme stability assay S27

10. Serum stability assay S27

11. *In vitro* anti-virus assays S28

11.1 Inhibition of pseudovirus infection S28

11.2 Inhibition of authentic coronavirus infection S28

11.3 Inhibition of S protein-mediated cell-cell fusion S29

11.4 Cytotoxicity assay S29

12. *In vivo* assays S29

12.1 Evaluation of the in vivo protective activity of **XY4-C7** S30

12.2 Mouse pharmacokinetic studies S30

13. References S31

14. ^{1}H and ^{13}C NMR spectra of sulfonyl-γ-AA peptide building blocks S32

1. Synthesis of sulfonyl-γ-AApeptide building blocks

1.1 General Information

All chemicals and solvents were purchased and directly used without any purification from Fisher Scientific, Sigma-Aldrich, or Oakwood. Fmoc protected amino acids were purchased from Chem-Impex International. ^{1}H-NMR at 600 MHz and ^{13}C-NMR at 150 MHz using TMS as the internal standard were acquired from Inova 600 MHz NMR spectrometer. The mass of each building block was determined by high-resolution mass spectrometry detected by High-resolution MS (HRMS) on Agilent 6540 LC/QTOF.

1.2 Synthesis

The sulfonyl-γ-AApeptide building blocks were synthesized based on a previous report[1] and Fmoc-protected amino acids were used as the initial starting materials. The building blocks were synthesized using one of the following three routes, depending on the protective groups used (Scheme S1). Building blocks **BB1-BB4** were synthesized by route A. **BB5** and **BB6** were synthesized by route B and **BB7-BB11** were synthesized by route C.

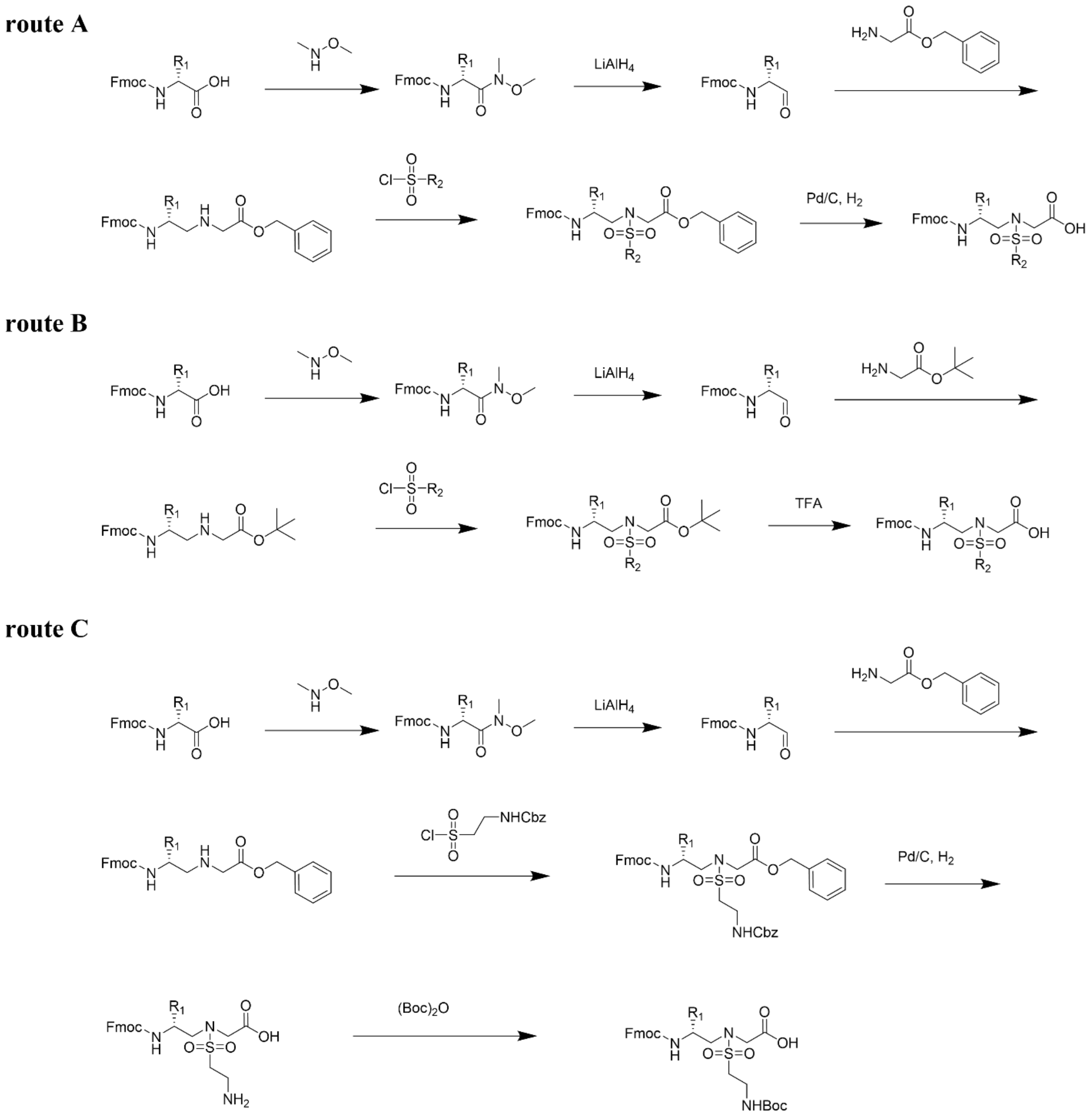

Scheme S1. General routes of synthesis of sulfonyl-γ-AApeptide building blocks.

1.3 Characterization of sulfonyl-γ-AApeptide building blocks

(S)-N-(2-((((9H-fluoren-9-yl)methoxy)carbonyl)amino)-3-(tert-butoxy)propyl)-N-(methylsulfonyl)glycine (BB7). ^{1}H NMR (600 MHz, Chloroform-*d*) δ 7.76 (d, *J* = 7.5 Hz, 2H), 7.60 (dd, *J* = 7.6, 4.4 Hz, 2H), 7.40 (t, *J* = 7.5 Hz, 2H), 7.34 – 7.30 (m, 2H), 4.42 (dd, *J* = 10.6, 7.2 Hz, 1H),

4.36 – 4.30 (m, 2H), 4.23 (t, J = 7.2 Hz, 1H), 4.15 – 4.10 (m, 1H), 3.95 (d, J = 9.1 Hz, 1H), 3.55 – 3.47 (m, 2H), 3.40 (ddd, J = 20.3, 12.1, 5.2 Hz, 2H), 2.99 (s, 3H), 1.19 (s, 9H). ^{13}C NMR (150 MHz, Chloroform-*d*) δ 172.82, 156.66, 143.74, 141.29, 127.75, 127.14, 125.23, 120.01, 73.76, 67.19, 60.96, 49.13, 48.67, 48.16, 47.09, 39.59, 27.43. HRMS (ESI) ([M+H]$^+$) Calcd. for $C_{25}H_{32}N_2O_7S$: 505.1930, found: 505.1927.

(R)-N-(2-((((9H-fluoren-9-yl)methoxy)carbonyl)amino)propyl)-N-((2-((tert-butoxycarbony-l)amino)ethyl)sulfonyl)glycine (BB8). ^{1}H NMR (600 MHz, Chloroform-*d*) δ 7.76 (d, J = 7.6 Hz, 2H), 7.61 (t, J = 6.9 Hz, 2H), 7.40 (t, J = 7.5 Hz, 2H), 7.32 (t, J = 7.5 Hz, 2H), 7.26 (s, 1H), 4.41 (dd, J = 10.4, 7.2 Hz, 1H), 4.31 (d, J = 20.5 Hz, 2H), 4.23 (t, J = 7.4 Hz, 1H), 3.94 (s, 1H), 3.73 – 3.55 (m, 3H), 3.37 (d, J = 11.6 Hz, 1H), 3.31 (s, 2H), 3.25 – 3.19 (m, 1H), 1.49 (d, J = 6.3 Hz, 3H), 1.44 (d, J = 3.1 Hz, 9H). ^{13}C NMR (150 MHz, Chloroform-*d*) δ 171.99, 156.46, 143.80, 141.28, 127.71, 127.14, 125.33, 125.23, 119.97, 80.66, 67.05, 60.53, 52.08, 47.10, 44.41, 35.33, 28.36, 21.10, 18.59. HRMS (ESI) ([M+H]$^+$) Calcd. for $C_{27}H_{35}N_3O_8S$: 562.2145, found: 562. 2138.

(R)-N-(2-((((9H-fluoren-9-yl)methoxy)carbonyl)amino)-4-methylpentyl)-N-((2-((tert-butox-ycarbonyl)amino)ethyl)sulfonyl)glycine (BB10). ^{1}H NMR (600 MHz, Chloroform-*d*) δ 7.75 (dd, J = 7.6, 2.3 Hz, 2H), 7.61 (dd, J = 11.2, 7.3 Hz, 2H), 7.39 (t, J = 7.4 Hz, 2H), 7.31 (t, J = 7.5 Hz, 2H), 7.26 (s, 1H), 4.94 (dd, J = 26.6, 9.4 Hz, 1H), 4.45 (dd, J = 10.6, 6.9 Hz, 1H), 4.36 – 4.21 (m, 3H), 3.92 (s, 1H), 3.68 – 3.56 (m, 2H), 3.33 (t, J = 7.7 Hz, 1H), 3.28 (td, J = 13.5, 11.0, 7.3 Hz, 2H), 3.21 (d, J = 17.9 Hz, 1H), 1.67 (m, 1H), 1.46 (d, J = 34.3 Hz, 11H), 0.92 (t, J = 7.0 Hz, 6H). ^{13}C NMR (150 MHz, Chloroform-*d*) δ 172.09, 156.81, 143.74, 141.30, 127.68, 127.13, 125.22, 124.65, 119.94, 80.68, 66.95, 60.58, 52.90, 51.61, 47.21, 41.84, 35.34, 28.36, 24.81, 23.25, 21.96. HRMS (ESI) ([M+H]$^+$) Calcd. for $C_{30}H_{41}N_3O_8S$: 604.2614, found: 604.2599.

Other building blocks are characterized in our previous works[1].

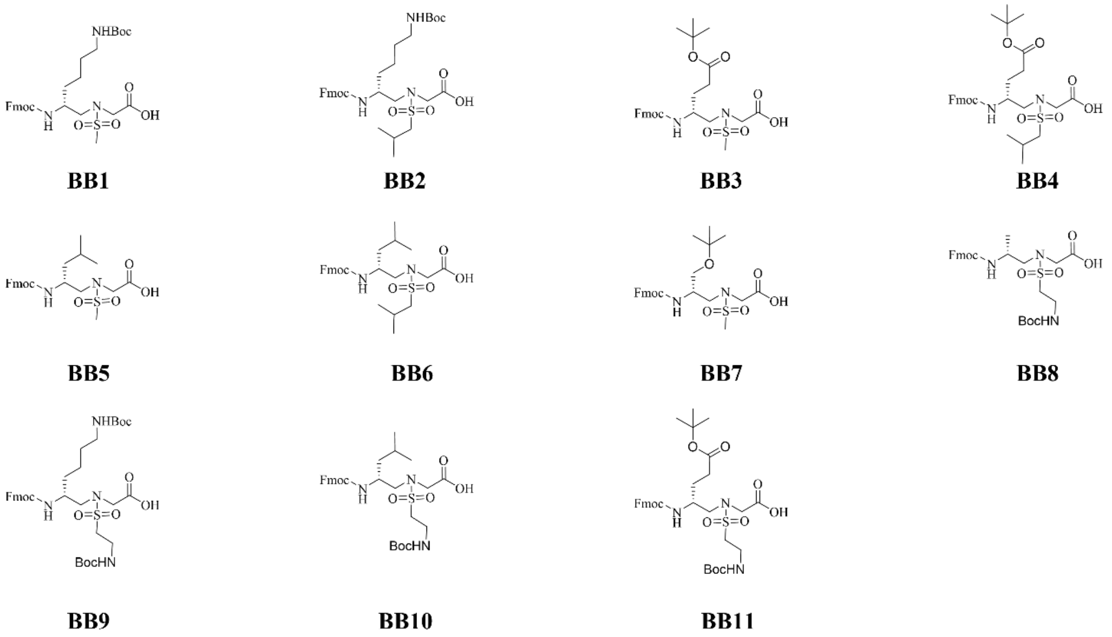

Figure S1. Structure of sulfonyl-γ-AApeptide building blocks.

2. Synthesis of Chol-PEGn-bromoacetate

The Chol-PEGn were synthesized according to the previous report[2] and the cholesterol were used as starting material. Chol-PEG4, Chol-PEG8, Chol-PEG12, and Chol-PEG24 were synthesized using the following routes (Scheme S2). Chol-PEGn-bromoacetate were also synthesized based on the previous report[3] and using the following routes. Briefly, a mixture of Chol-PEG4, Chol-PEG8, Chol-PEG12, or Chol-PEG24 with bromoacetic acid was dissolved in CH_2Cl_2. Then 2 eq of N,Ndiisopropylcarbodiimide (DIPEA) and 0.01 eq of 4-dimethylaminopyridine (DMAP) were added. The solution was left stirring at room temperature for 48 h and analyzed by TLC. Afterward, the solvent was removed under vacuum, and the crude was purified by flash column chromatography on silica gel as a white solid.

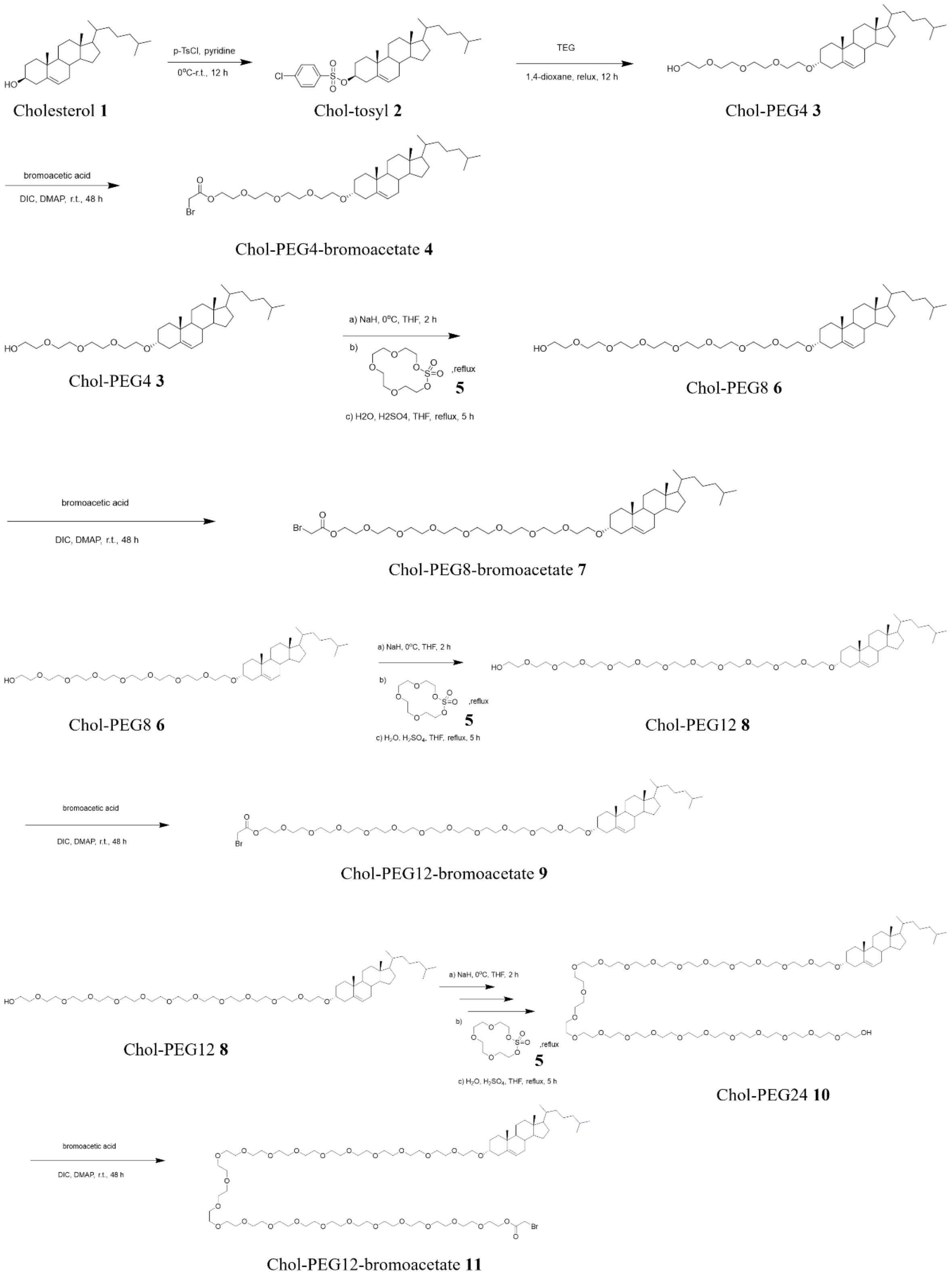

Scheme S2. General routes of synthesis of Chol-PEGn-bromoacetate.

3. Preparation of sulfonyl-γ-AA peptide sequences mimicking the HR2 peptide on the fusion core

3.1 General Information

Solid phase synthesis was carried out in the peptide synthesis vessels on the Burrell Wrist-Action shaker. Rink amide-MBHA resin (0.6 mmol/g) was used for the synthesis of sulfonyl-γ-AApeptides. All peptides were analyzed and purified using a method that involved a 5-100% linear gradient of solvent B (0.1% TFA in acetonitrile) in solvent A (0.1% TFA in H_2O) over a 50-minute period, followed by 100% solvent B over a 15-minute period, on a Waters Breeze 2 HPLC system equipped with both analytic column (1 mL/min) and preparative column (16 mL/min). A Labconco freeze-drying machine was used to lyophilize purified sequences. The mass of each sulfonyl-γ-AApeptides sequence was confirmed with MALDI on Applied Biosystems 4700 Proteomics Analyzer.

3.2 Synthesis

3.2.1 Synthesis of sulfonyl-γ-AApeptides

Sulfonyl-γ-AA peptide sequences were synthesized based on our previous reports[4] and followed the route in Scheme S3. Solid phase synthesis was started from 100 mg Rink Amide-MBHA resin (0.6 mmol/g) under room temperature at atmospheric pressure. The resin was first swelled by soaking in DMF for 10 min, then the Fmoc group was deprotected by shaking in 20% piperidine/DMF (15 min × 2), and then the resin was washed three times with DCM and three times with DMF. The coupling reaction was finished by adding a premixed solution of the Fmoc-protected regular amino acid/sulfonyl-γ-AApeptide building block (2 eq), HOBt (4 eq), and DIC (4 eq) in 3 mL DMF to the resin and shaking for 4 h. The resin was treated with 20%

piperidine/DMF solution (15 min × 2) after being washed with DCM and DMF. Following the same coupling procedure, another Fmoc-protected regular amino acid/sulfonyl-γ-AApeptide building block was attached to the resin. The reaction cycles were carried out repeatedly until the desired sulfonyl-γ-AApeptides were synthesized. After the N-terminus of the sulfonyl-γ-AApeptide sequences was acetylated by acetic anhydride (1 mL) in pyridine (2 mL) for 15 min, the desired sequences were cleaved from the resin by treating with 1:1 TFA/DCM solution (4 mL, 2h). The resin was washed with DCM three times after the cleavage solution was collected. The cleavage solution and the wash solutions were combined, and the resulting crude product was vacuum dried. The crude sequences were analyzed and purified on a Waters HPLC system. All the HR2 fusion core mimic sulfonyl-γ-AApeptides were obtained at > 95% purity after prep-HPLC purification.

3.2.2 Synthesis of Chol-PEGn-sulfonyl-γ-AApeptides

The synthesis of Chol-PEGn- sulfonyl-γ-AApeptides was based on the previous reported[5]. Briefly, Chol-PEGn-bromoacetate (1 eq) in CH_3CN was added to a solution of purified sulfonyl-γ-AApeptide sequence in 30 mM NH_4HCO_3 buffer. The mixture was stirred at room temperature for 4 h. Acetic acid was subsequently added dropwise to pH 5 under ice cooling. Chol-PEGn-sulfonyl-γ-AApeptides were purified by preparative reverse phase HPLC to afford the compound as a white powder after lyophilization.

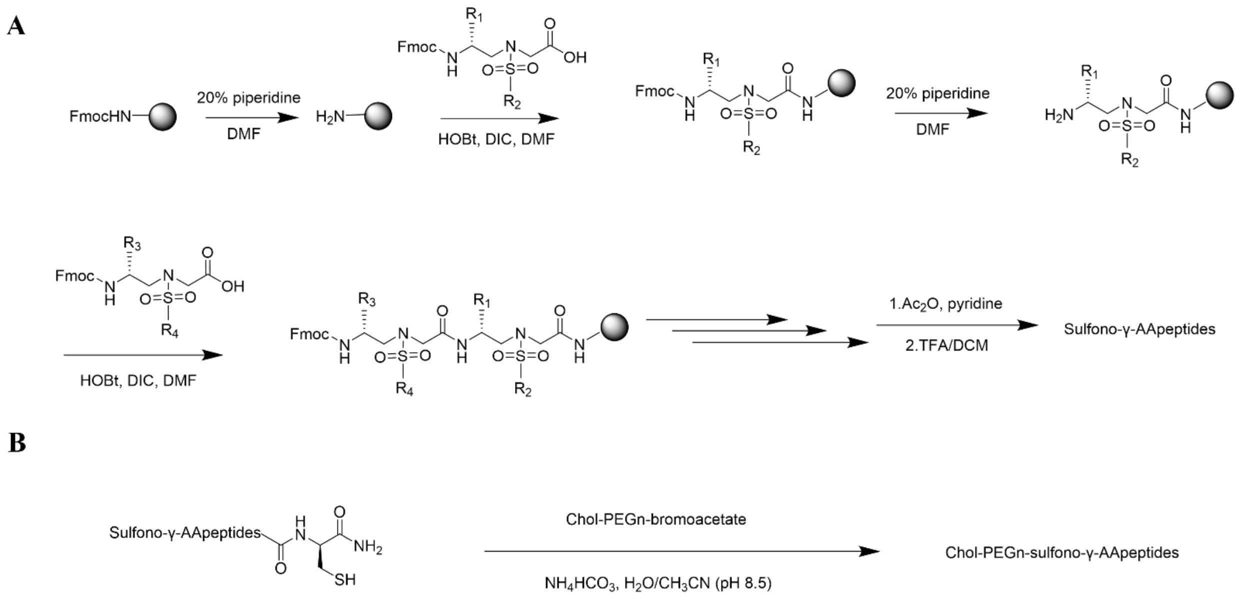

Scheme S3. General routes of synthesis of sulfonyl-γ-AApeptides **(A)**, and Chol-PEGn- sulfonyl-γ-AApeptides **(B)**.

3.3 Characterization of sulfonyl-γ-AApeptides and Chol-PEGn-sulfonyl-γ-AApeptides

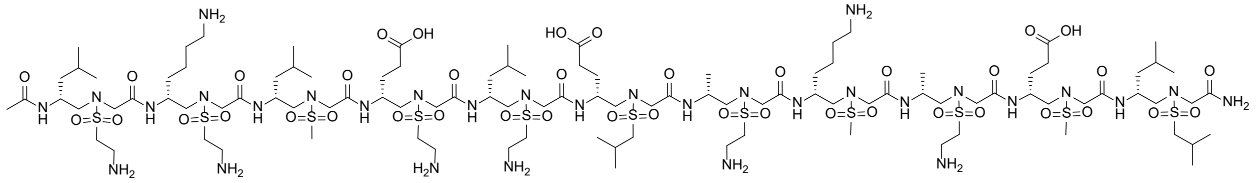

XY1

Chemical Formula: $C_{104}H_{211}N_{31}O_{40}S_{11}$

Theoretical Mol. Wt: 2888.6690

Observed (MALDI-TOF): 2889.5367 ($M+H^{+}$)

Purity: 98.62%

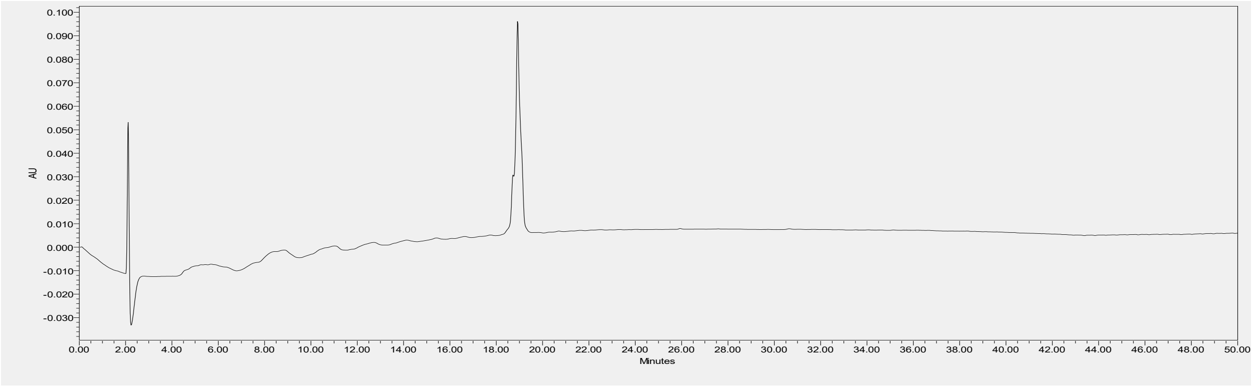

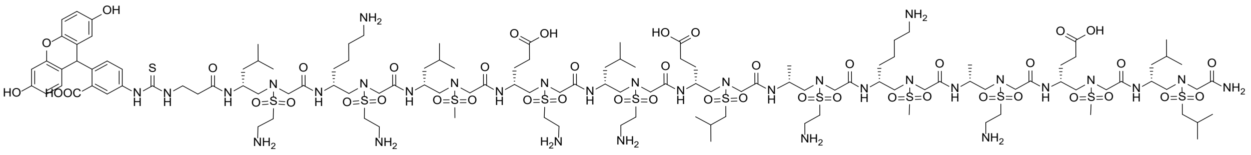

FITC-XY1

Chemical Formula: $C_{126}H_{227}N_{33}O_{45}S_{12}$

Theoretical Mol. Wt: 3309.1080

Observed (MALDI-TOF): 3332.1123 ($M+Na^{+}$)

Purity: 98.02%

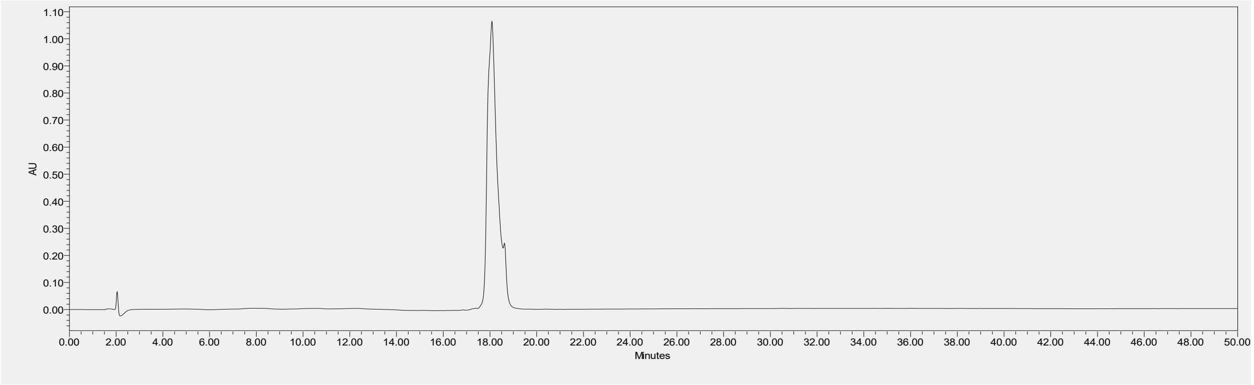

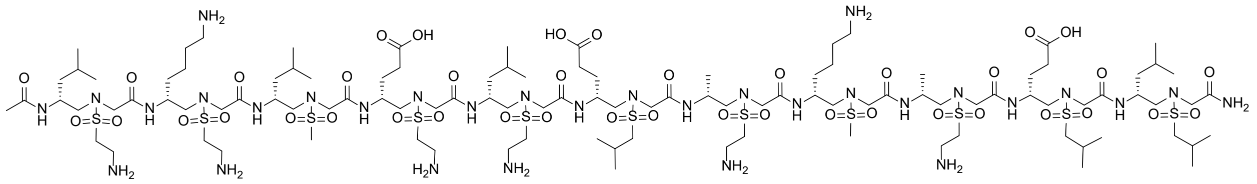

XY2

Chemical Formula: $C_{107}H_{217}N_{31}O_{40}S_{11}$

Theoretical Mol. Wt: 2930.7500

Observed (MALDI-TOF): 2931.4500 (M+H$^+$)

Purity: 99.33%

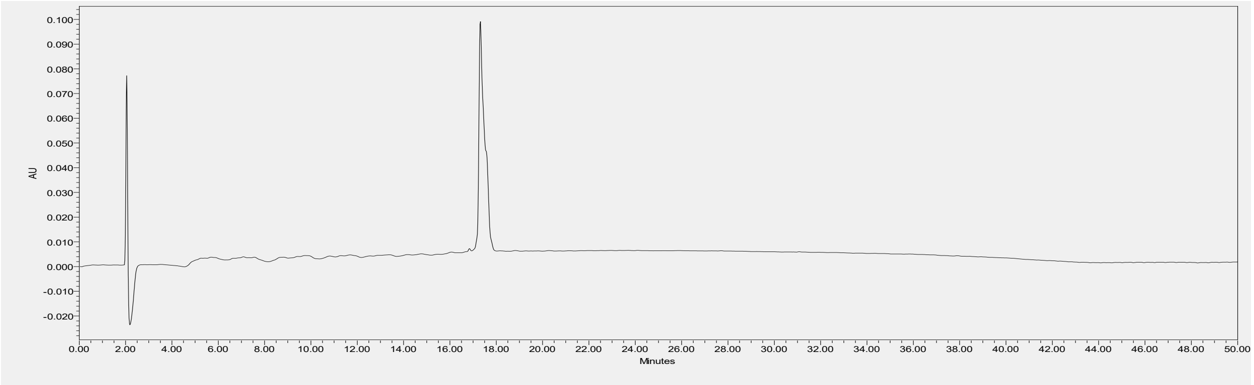

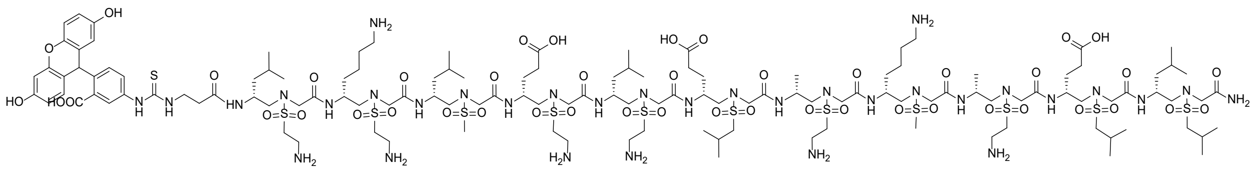

FITC-XY2

Chemical Formula: $C_{129}H_{233}N_{33}O_{45}S_{12}$

Theoretical Mol. Wt: 3351.1890

Observed (MALDI-TOF): 3391.1580 (M+K$^+$)

Purity: 99.16%

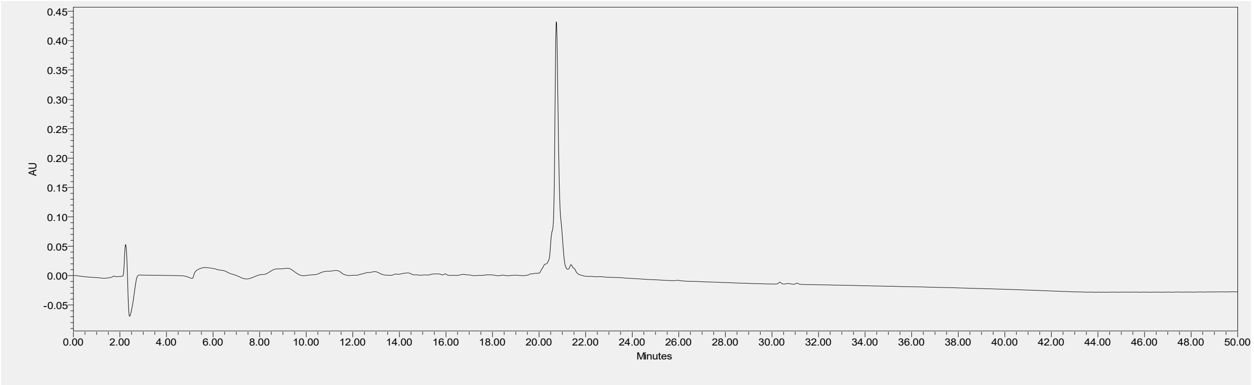

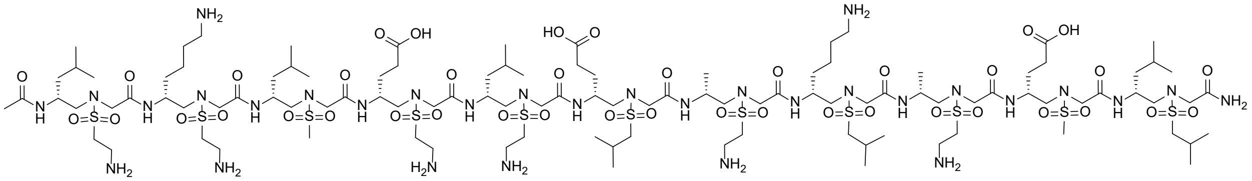

XY3

Chemical Formula: $C_{107}H_{217}N_{31}O_{40}S_{11}$

Theoretical Mol. Wt: 2930.7500

Observed (MALDI-TOF): 2931.6553 ($M+H^+$)

Purity: 99.58%

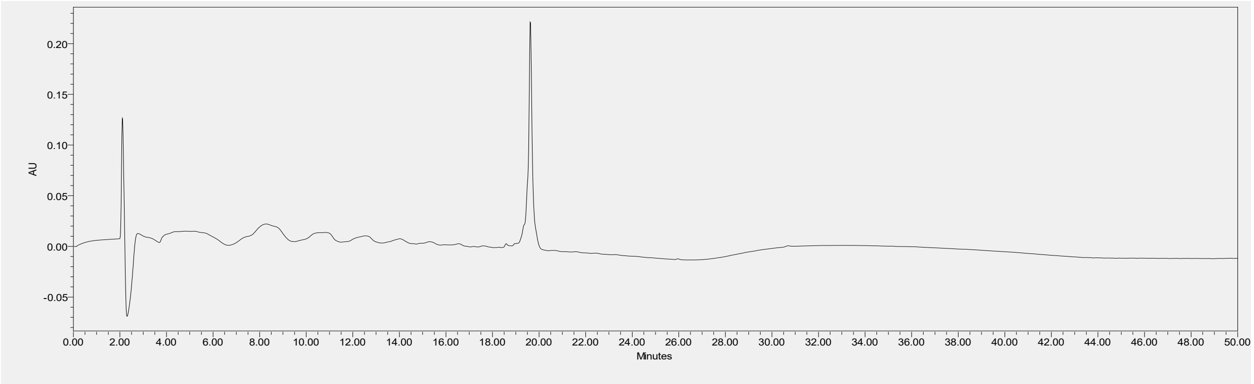

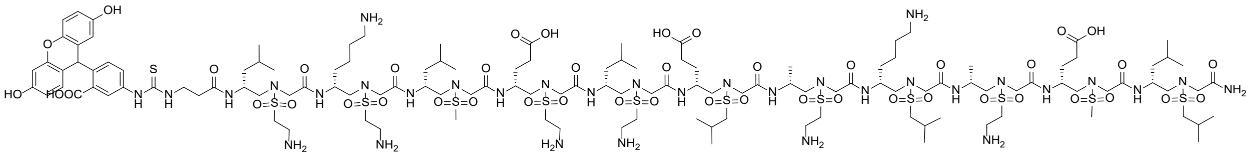

FITC-XY3

Chemical Formula: $C_{129}H_{233}N_{33}O_{45}S_{12}$

Theoretical Mol. Wt: 3351.1890

Observed (MALDI-TOF): 3352.2220 ($M+H^+$)

Purity: 99.90%

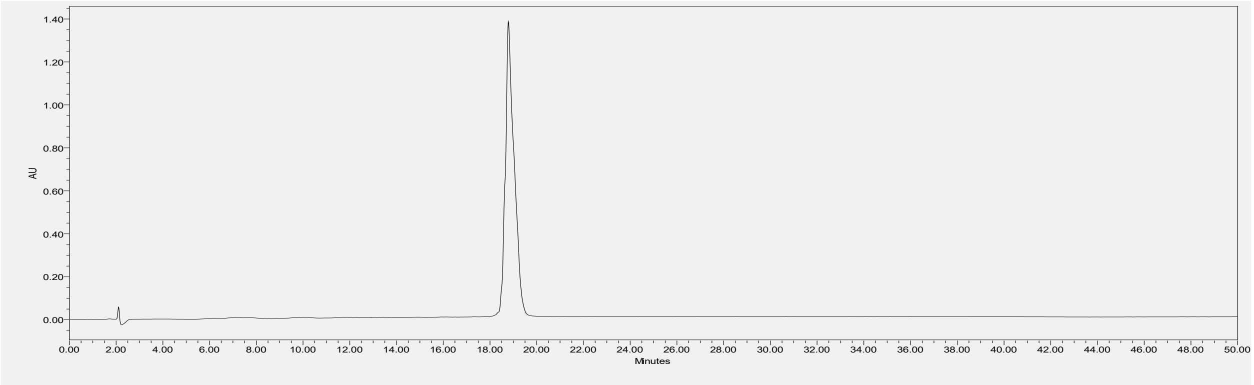

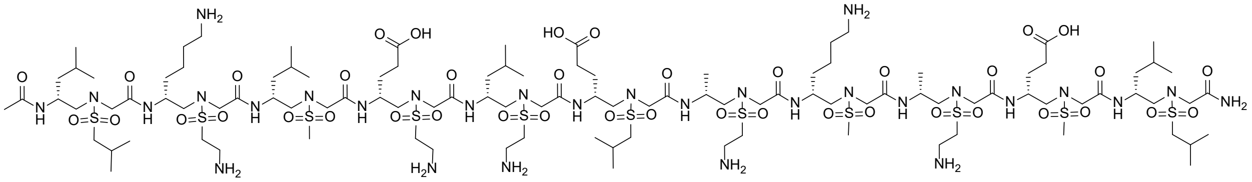

XY4

Chemical Formula: $C_{106}H_{214}N_{30}O_{40}S_{11}$

Theoretical Mol. Wt: 2901.7080

Observed (MALDI-TOF): 2940.7300 ($M+K^+$)

Purity: 99.98%

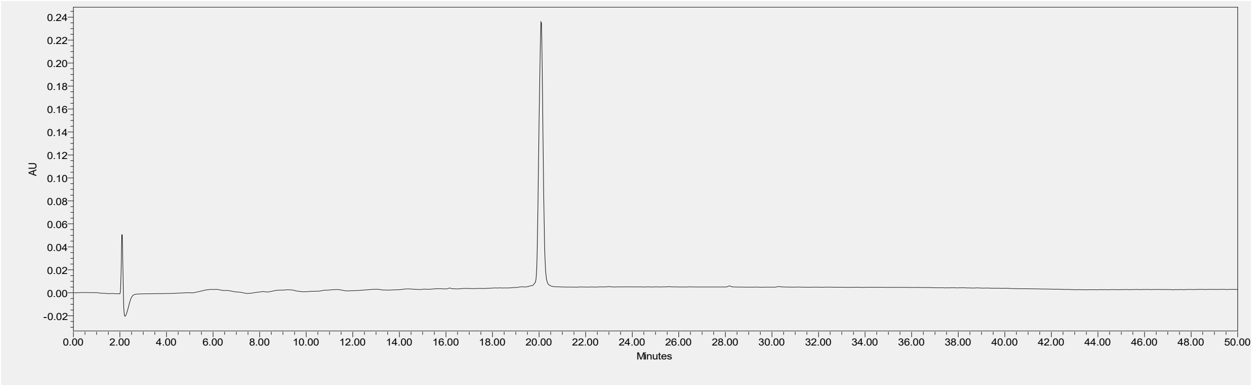

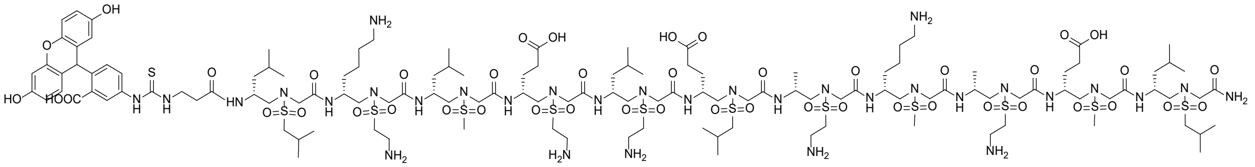

FITC-XY4

Chemical Formula: $C_{128}H_{230}N_{32}O_{45}S_{12}$

Theoretical Mol. Wt: 3322.1470

Observed (MALDI-TOF): 3323.1560 ($M+H^+$)

Purity: 99.88%

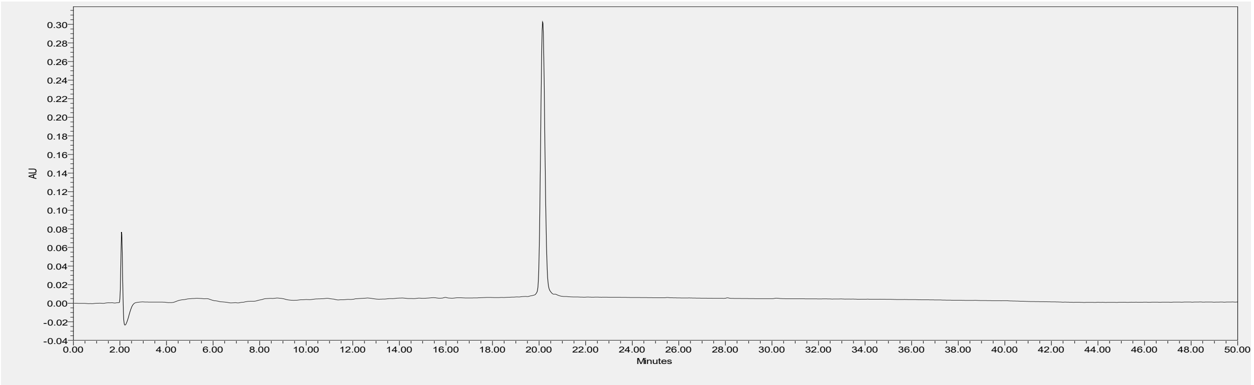

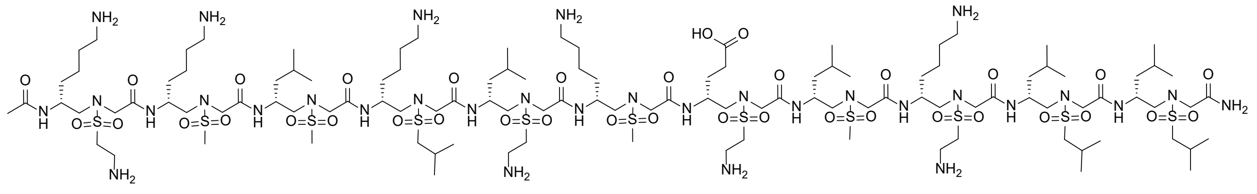

XY5

Chemical Formula: $C_{113}H_{234}N_{32}O_{36}S_{11}$

Theoretical Mol. Wt: 2969.9630

Observed (MALDI-TOF): 2970.8878 ($M+H^{+}$)

Purity: 99.19%

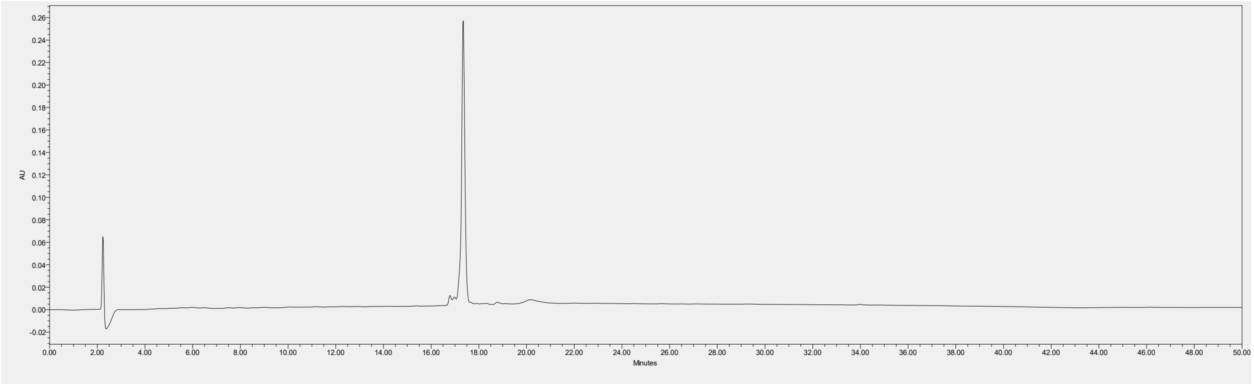

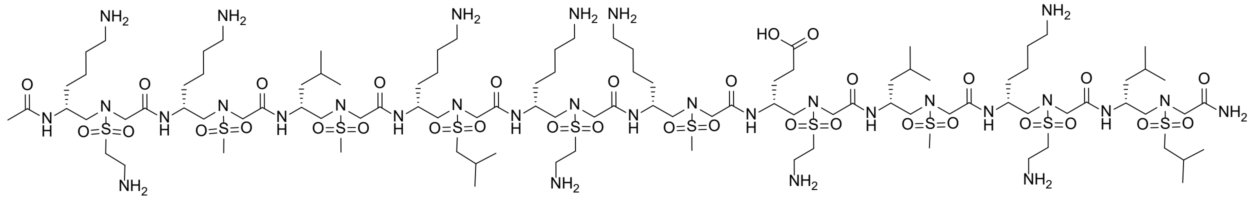

XY6

Chemical Formula: $C_{101}H_{211}N_{31}O_{33}S_{10}$

Theoretical Mol. Wt: 2708.5830

Observed (MALDI-TOF): 2709.5950 (M+H$^+$)

Purity: 99.50%

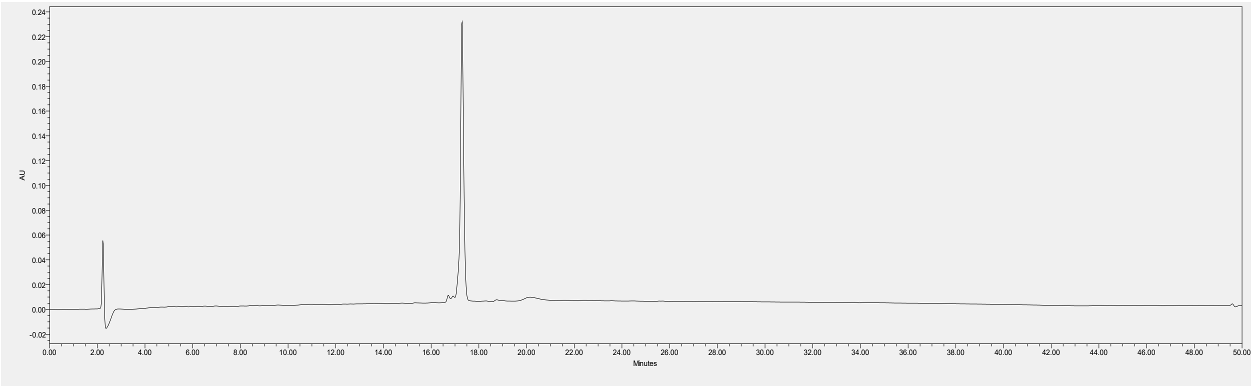

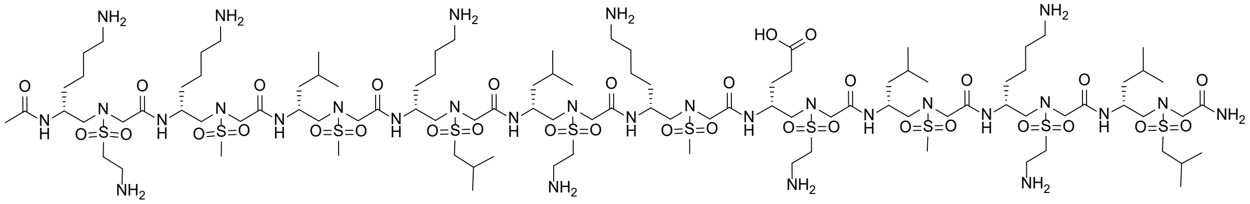

XY7

Chemical Formula: $C_{101}H_{210}N_{30}O_{33}S_{10}$

Theoretical Mol. Wt: 2693.5680

Observed (MALDI-TOF): 2694.5655 (M+H$^+$)

Purity: 99.35%

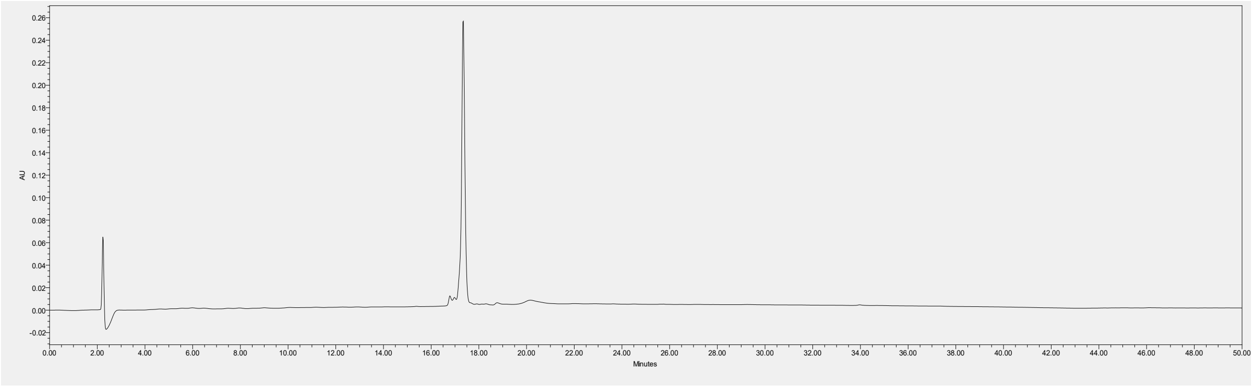

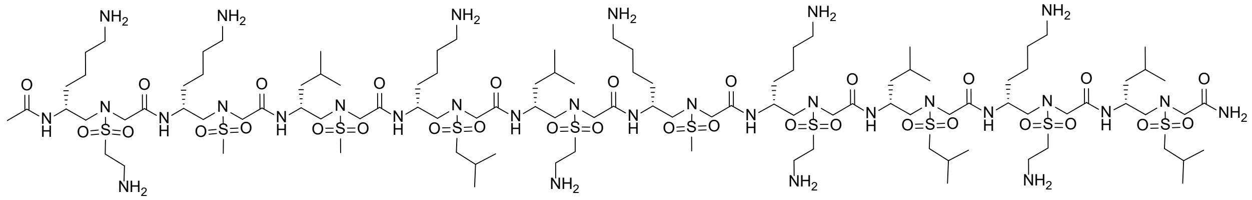

XY8

Chemical Formula: $C_{105}H_{221}N_{31}O_{31}S_{10}$

Theoretical Mol. Wt: 2734.7090

Observed (MALDI-TOF): 2735.7111 (M+H^{+})

Purity: 99.75%

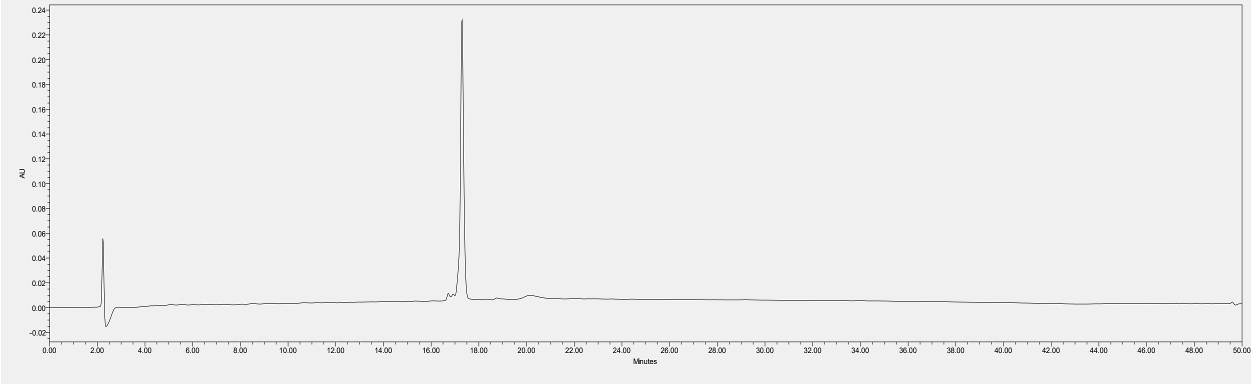

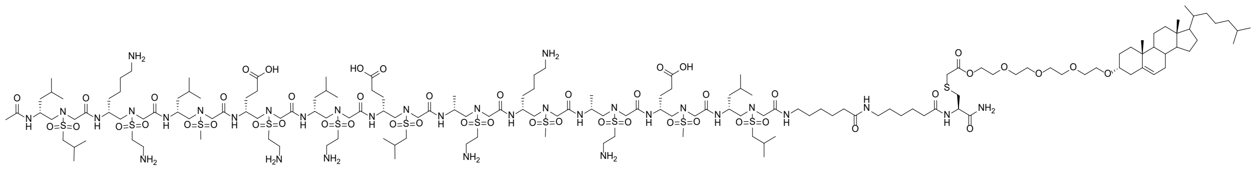

XY4-C1

Chemical Formula: $C_{158}H_{303}N_{33}O_{49}S_{12}$

Theoretical Mol. Wt: 3830.8881

Observed (MALDI-TOF): 3853.4200 ($M+Na^{+}$)

Purity: 98.58%

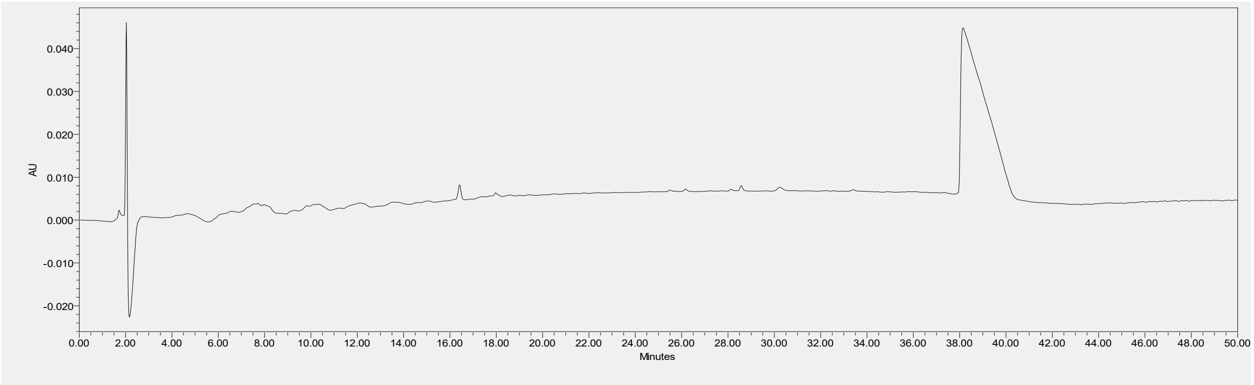

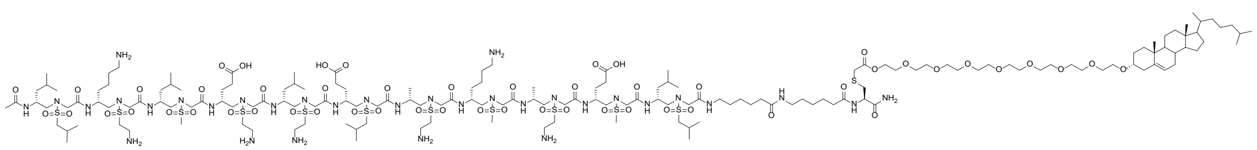

XY4-C2

Chemical Formula: $C_{166}H_{319}N_{33}O_{53}S_{12}$

Theoretical Mol. Wt: 4006.9930

Observed (MALDI-TOF): 4029. 7800 ($M+Na^{+}$)

Purity: 99.00%

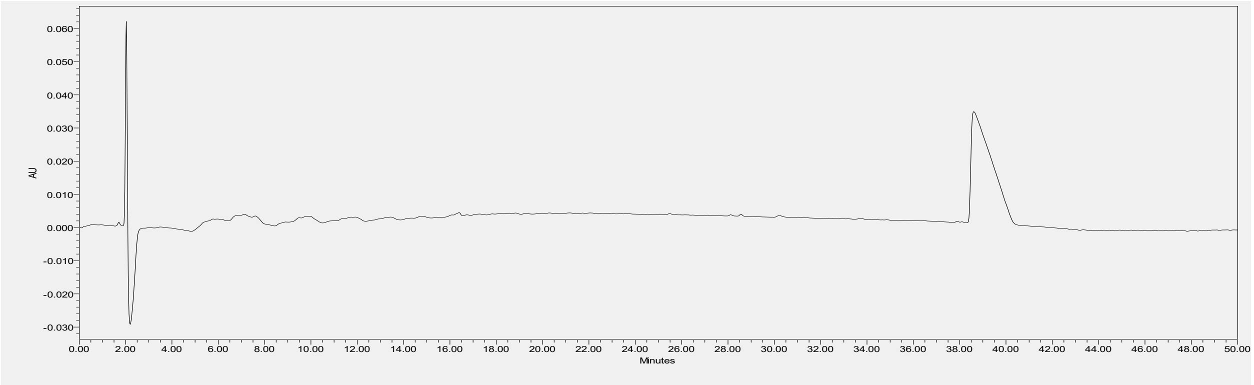

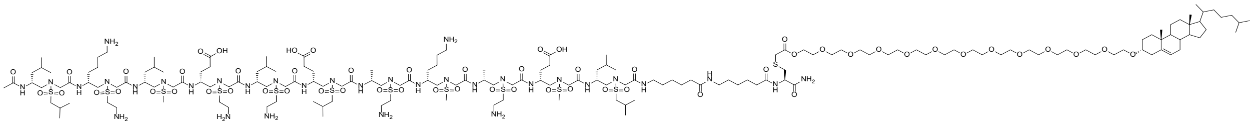

XY4-C3

Chemical Formula: $C_{174}H_{335}N_{33}O_{57}S_{12}$

Theoretical Mol. Wt: 4186.4880

Observed (MALDI-TOF): 4209.9200 ($M+Na^+$)

Purity: 99.32%

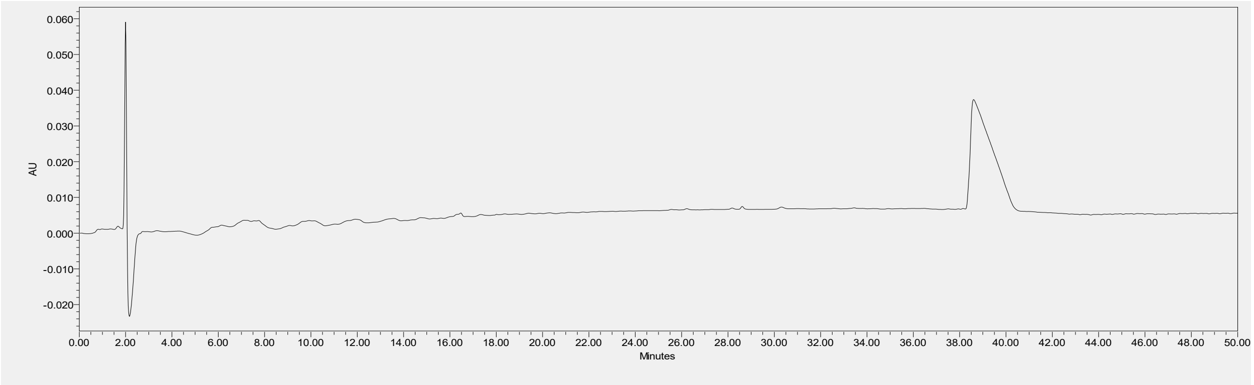

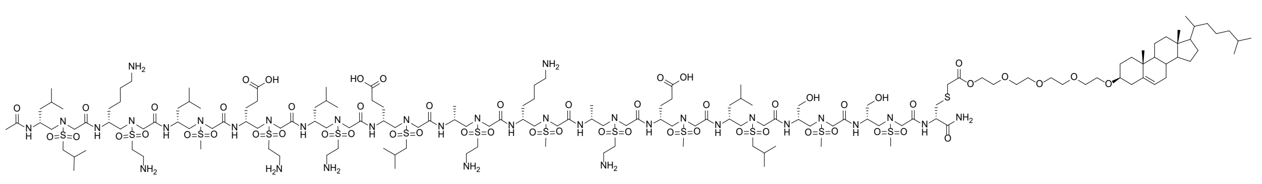

XY4-C4

Chemical Formula: $C_{158}H_{305}N_{35}O_{55}S_{14}$

Theoretical Mol. Wt: 4024.2080

Observed (MALDI-TOF): 4025.4000 ($M+H^{+}$)

Purity: 98.79%

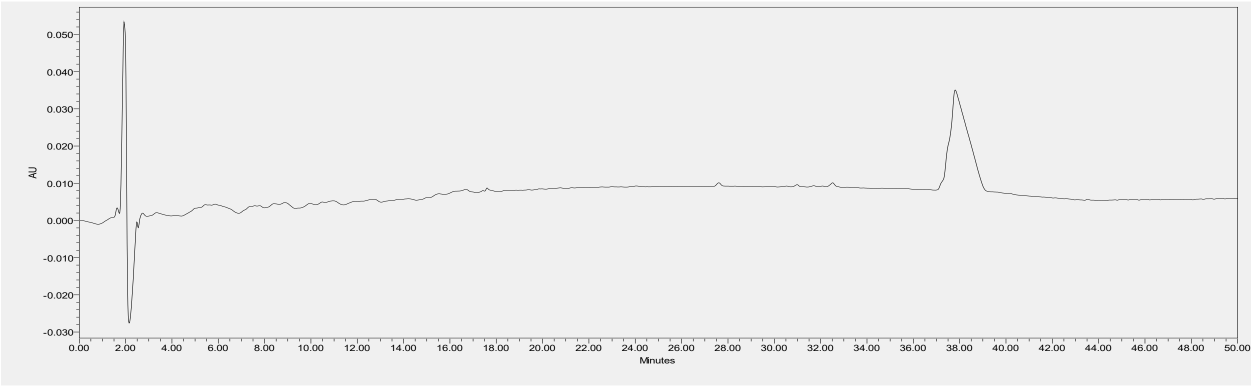

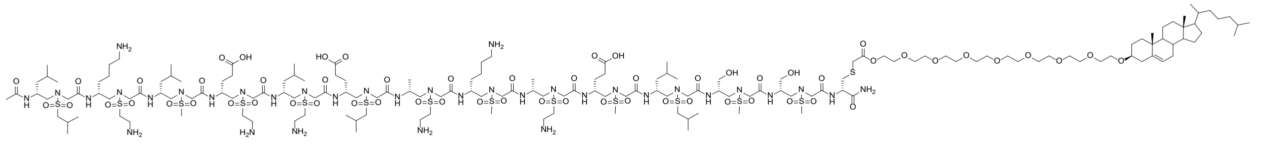

XY4-C5

Chemical Formula: $C_{166}H_{321}N_{35}O_{59}S_{14}$

Theoretical Mol. Wt: 4196.9284

Observed (MALDI-TOF): 4197.2000 ($M+H^{+}$)

Purity: 98.11%

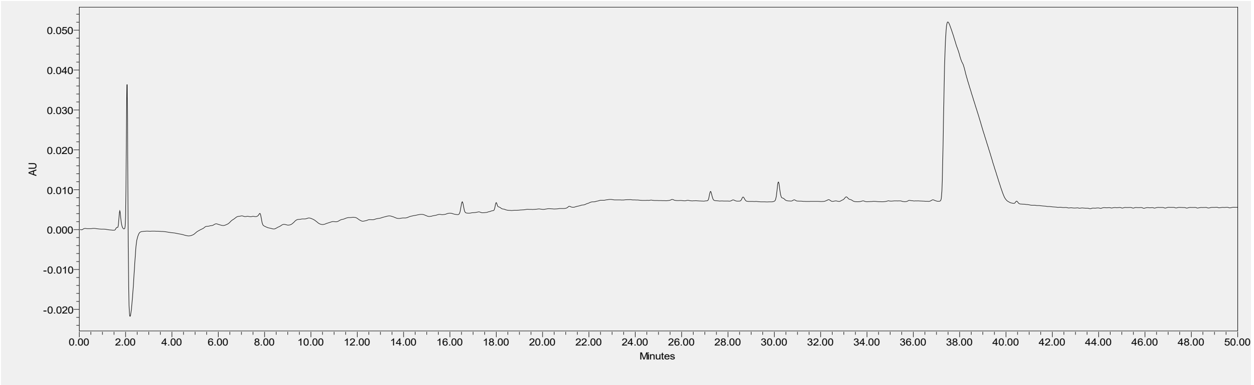

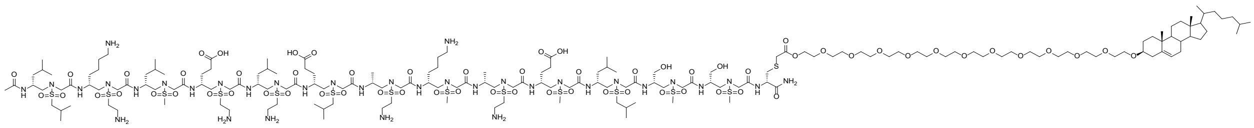

XY4-C6

Chemical Formula: $C_{174}H_{337}N_{35}O_{63}S_{14}$

Theoretical Mol. Wt: 4376.6320

Observed (MALDI-TOF): 4377.0612 ($M+H^+$)

Purity: 98.50%

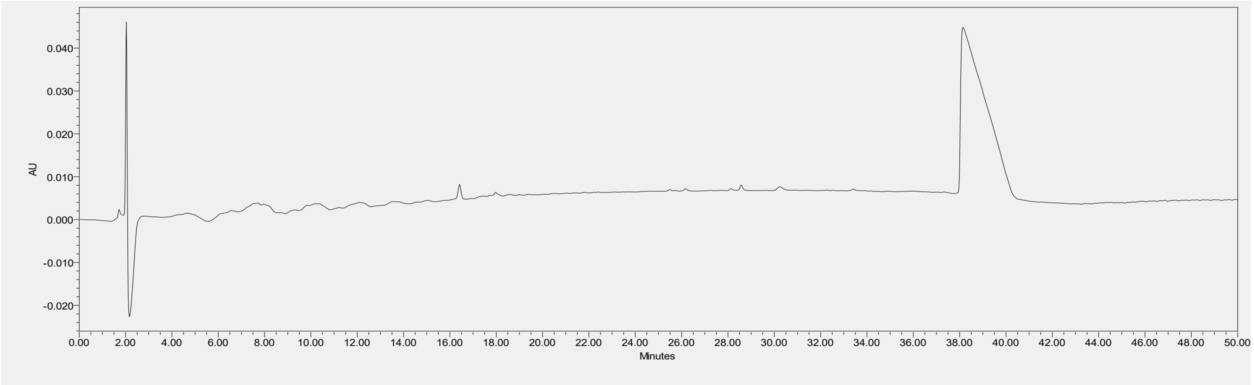

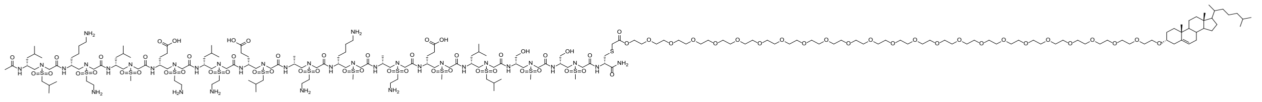

XY4-C7

Chemical Formula: $C_{198}H_{385}N_{35}O_{75}S_{14}$

Theoretical Mol. Wt: 4905.2680

Observed (MALDI-TOF): 4906.1606 ($M+H^{+}$)

Purity: 99.45%

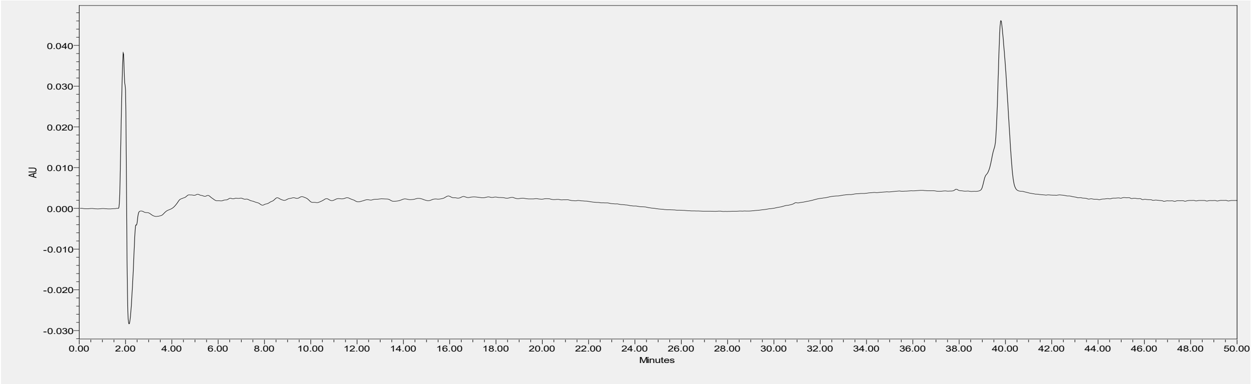

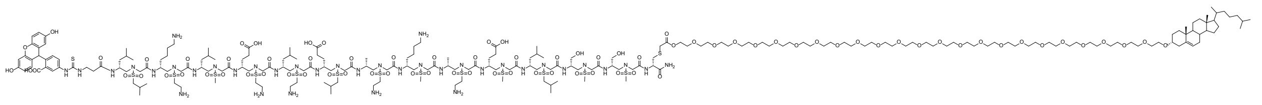

FITC-XY4-C7

Chemical Formula: $C_{220}H_{401}N_{37}O_{80}S_{15}$

Theoretical Mol. Wt: 5325.7070

Observed (MALDI-TOF): 5348.9414 ($M+Na^{+}$)

Purity: 99.36%

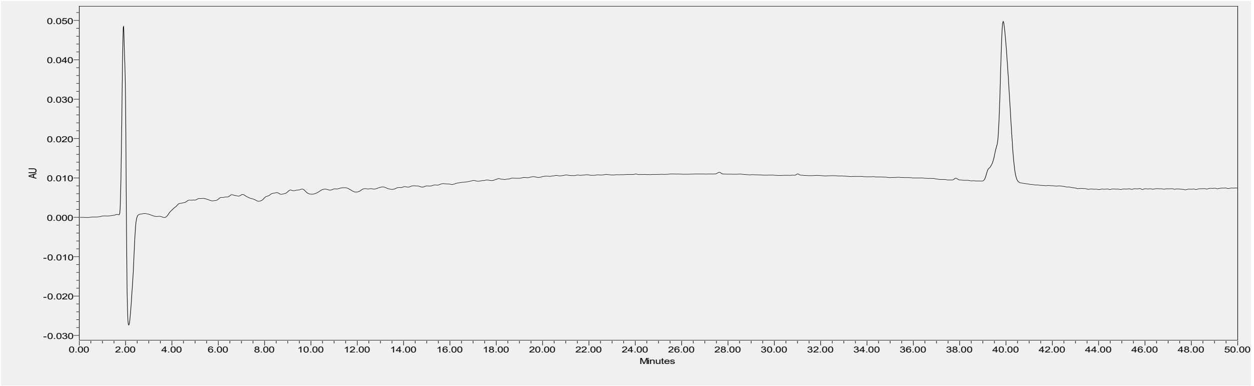

4. Fluorescence polarization assay[6]

Proteins (0-2 μM) in PBS were treated with 50 nM FITC were treated with 50 nM FITC-labeled γ-AApeptides. Fluorescence anisotropy values were plotted as a function of protein concentration to determine the dissociation onstants (Kd), and the graphs were then fitted to the following equation.

$$y = [FPmin + (FPmax - FPmin)]$$

$$\frac{(K\mathrm{d} + Lst + x) - \sqrt{(K_d + L_{st} + x)^2 - 4L_{st} \times x}}{2L_{st}}$$

L_{st} and x refer to the concentration of the peptide and protein, respectively. The experiments were conducted in triplicate and repeated three times.

5. Circular Dichroism Spectroscopy

With the use of an Aviv 215 spectrometer in a 1 mm path quartz cuvette, CD spectra of samples were obtained in PBS at 100 μM concentrations[4]. An average of 3 scans for each sample run was taken thrice and averaged. The PBS sample that was used as blank was evaluated the same method

and subtracted from average sample readings. The equation below was used to calculate molar ellipticity [θ].

$[\theta] = \theta_{obs} / (n\times l \times c \times 10)$

θ_{obs} = measured ellipticity in millidegrees

n = number of side chains

l = path length in centimeter (0.1 cm)

c = concentration of samples (M)

CD spectra of mechanism was recorded by previous method[7]. The HR1 peptide (911-987, NovoPep Limited), the HR2 peptide (1175-1201, synthesized) and **XY4-C7** were dissolved in PBS buffer at a final concentration of 40 μM. Briefly, HR1 peptides were mixed with HR2 helices at 37 ºC for 1 h. After that, added **XY4-C7** to further incubated for 30 min. The CD wave scans were measured by an Aviv 215 spectrometer from 190 to 260 nm.

The HR1 peptide (911-987):

TQNVLYENQKLIANQFNSAIGKIQDSLSSTASALGKLQDVVNQNAQALNTLVKQLSSNF GAISSVLNDILSRLDKVE

The HR2 peptide (1175-1201):

SVVNIQKEIDRLNEVAKNLNESLIDLQ

6. PAMPA-BBB assay

The PAMPA-BBB assay produce was developed by pION that followed by previous reported[6]. The TECAN Freedom EVO150 robot carried out each step of the liquid handling process, and

Pion PAMPA Evolution software was used to analyze the results. BBB PAMPA consisted of Stirwell^{TM}PAMPA Sandwich plate preloaded with magnetic stirring disks, brain the sink buffer (BSB), and lipid solution (BBB-1). 200 μL of BSB (pH 7.4) was added to the acceptor well after 4 μL of lipid solution had been put there. 180 μL of diluted **XY4-C7** (50-250 μM in system buffer at pH 7.4 from a 10 mM DMSO stock solution) was then added to the donor well. The PAMPA sandwich plate was assembled, placed on the Gut-Box^{TM} and stirred with 60 μm Aqueous Boundary Layer (ABL) settings for 1 h incubation. The distribution of compounds in the donor and acceptor buffers (150 μL aliquot) was determined using the TECAC S20Infinnite M-1000 Pro microplate reader to measure UV spectra from 250 to 498 nm. Permeability (P_{app}, 10^{-6} cm/s) of each compound was calculated by Pion PAMPA evolution software. The assay was performed in triplicate.

7. PAMPA-GIT assay[6]

The PAMPA-GIT assay was also carried out utilizing a pION-developed technique. All liquid handling procedures were performed using the TECAC Freedom EVO 150 robot, and the data was evaluated using pION's PAMPA Evolution software. The acceptor sink buffer (ASB), GIT-0 Lipid solution, and the StirwellTM PAMPA sandwich plate preloaded with magnetic disks are all included in the pION's GIT PAMPA. In the acceptor well, 4 μL of lipid were transferred, followed by 200 μL of ASB (pH 7.4). The donor wells were then filled with 180 μL of diluted test compound (50-250 μM in system buffer at pH 5.0, 6.2, and 7.4 from a 10 mM DMSO solution). The PAMPA sandwich plate was put together and positioned on the Gur-BoxTM. It was then agitated for 30 min at 40 μm Aqueous Boundary Layer (ABL) settings. The distribution of the compounds in the donor and acceptor buffers (150 μL aliquot) was determined by UV spectra measurement from 250 to 498 nm using the TECAN Infinite M-1000 Pro microplate reader. Then the Permeability

(P_{app}, 10^{-6}cm/s) of each compound was calculated by Pion PAMPA evolution software. The assay was performed in triplicate.

8. ITC assay

The interaction between **XY4-C7** and the HR1 peptide was determined using an ITC microcalorimeter instrument. In brief, the HR1 peptide was dissolved in ACN/H_2O to 10 μM before being injected into the chamber containing 100 μM of **XY4-C7**. Titration was carried out at a constant stirring speed of 220 rpm and temperature of 23 °C. Twenty-five injections were automatically performed with an injection interval of 180 sec. After that, iTC-200 software was used to analyze the data.

9. Enzyme stability assay[6]

XY4-C7 or the HR2 peptide (0.1 mg/mL) was incubated with 0.1 mg/mL pronase in 100 mM ammonium bicarbonate buffer (pH 7.8) at 37 °C for 24 h. Then, a speed vacuum was used to remove the water and ammonium bicarbonate from the reaction mixtures. The residues were dissolved in 100 μL ACN and analyzed by LC/MS/MS.

10. Serum stability assay[8]

The serum stability of **XY4-C7** or the HR2 peptide was determined in 50 % (v/v) aqueous serum from male AB plasma. **XY4-C7** or the HR2 peptide was dissolved in 30 μL DMSO, to which was added 970 μL of 50% aqueous pooled serum to make 1 mg/mL solution. The solution was incubated at 37 °C for 24 h. Subsequently, 100 μL of ACN was added to 100 μL of serum incubating solution on ice for 20 min, which was then centrifuged at 4 °C for 15 min. The supernatant should have a concentration at 0.5 mg/mL. A final concentration of 0.1 mg/mL can be

achieved by diluting 50 μL of supernatant with 200 μL H_2O. The vials containing diluted supernatants were then used for the LC/MS/MS analysis.

11. *In vitro* anti-virus assays

11.1 Inhibition of pseudovirus infection

The inhibitory activity of sulfonyl-γ-AApeptides was tested according to our previous pubicaitons[9, 10]. In our lab, we kept plasmids encoding the S protein of coronaviruses such as SARS-CoV-2, SARS-CoV, MERS-CoV, HCoV-NL63 and SARSr-CoV WIV1, luciferase reporter vectors (pNL4-3, Luc.R-E-). The pcDNA3.1-SARS-CoV-2-S and pNL4-3. Luc.R-E- were co-transfected into HEK-293T cells for the packaging of pseudoviruses using Vigofect transfection reagent, and the supernatants were then replaced with fresh media containing 10% FBS after eight hours. After 48 hours, the pseudovirus-containing supernatants were gathered, filtered using a 0.45 μm filter, and then stocked at -80 °C. Target cells were seeded at 8000 per well in a 96-well plate and grown at 37 °C for 12 h to ascertain a compound's inhibitory effect. The compound was diluted with DMEM without PBS, and then the same volume of pseudoviruses was added. The mixture (100 μL) was then added to the target cells and allowed to sit there for 30 minutes. The mixture was changed out to fresh medium after 12 h. After 48 h, the cells were lysed with cell lysis buffer, and the Luciferase Assay System was used to find luciferase activity (Promega, Madison, WI, USA).

11.2 Inhibition of authentic coronavirus infection

As previously reported[9, 10], the SARS-CoV-2 inhibition assay was carried out in a biosafety level 3 laboratory (BSL-3). The inhibitory activity of **XY4-C7** was evaluated against authentic viruses (SARS-CoV-2/SH01/human/2020/CHN). **XY4-C7** was serially diluted with DMEM without PBS. The diluted **XY4-C7** was combined with 100 TCID50 of the virus. The mixtures were transferred

to target cells (Caco2 cells or Vetro-E6 cells) after incubation for 30 minutes. The supernatants were collected after 48 h and the viral RNA load was tested as previously reported[10]. Briefly, the viral RNA was extracted with an RNA extraction kit (Transgene, China). Then the N gene of SARS-CoV-2 was evaluated by real-time RT-PCR. The sequence of primer and probe follows:

Forward: GGGGAACTTCTCCTGCTAGAAT;

Reverse: CAGACATTTTGCTCTCAAGCTG

Probe: 5′-FAM-TTGCTGCTGCTTGACAGATT-TAMRA-3′

11.3 Inhibition of S protein-mediated cell-cell fusion

The cell-cell fusion assay was performed as in previous reports[9, 10]. PAAV-IRES-EGFP S was transfected to HEK-293T cells to obtain effector cells expressing the S protein of SARS-CoV-2 and GFP. After that, serially diluted **XY4-C7** was mixed with effector cells (HEK-293T), and the mixture was transferred to target cells (Caco-2 cells). After incubation for 2-4 h, fused cells were counted, and the fusion rate was calculated to determine inhibitory activity.

11.4 Cytotoxicity assay

The cytotoxicity of **XY4-C7** to Caco-2 cells was tested as previously described[9, 10]. Briefly, we added the serially diluted compounds to the target cells (Caco-2 cells). The medium was changed to a fresh medium after culture at 37 °C for 12 h. The supernatant was removed after 48 h later and cell viability was analyzed with Cell Counting Kit (CCK-8; Dojindo, Kumamoto, Japan). 100 μL Cell Counting Kit-8 solution was added to each well in a 96-well plate and followed by an additional incubation for 2 h. The absorbance was measured at 450 nm.

12. *In vivo* assays

12.1 Evaluation of the *in vivo* protective activity of XY4-C7

The protective effect of **XY4-C7** against HCoV-OC43 *in vivo* was performed according to a previous study[9, 10]. The Institutional Laboratory Animal Care and Use Committee at Fudan University approved the use of animals in this study (Approval number: 20200821-002). Newborn mice infected with HCoV-OC43 were established as previously reported[9, 10]. Pregnant Balb/c mice (18 days) were separated into three groups after giving birth. Seven baby mice were split among each group. **XY4-C7** was intranasally administered at a dose of 1 mg/kg into mice in the preventive and therapeutic groups before or after the HCoV-OC43 challenge. Dissection of the newborn mice was done on the fourth post-infection day. The relative viral RNA expression level in the brain was evaluated through RT-PCR and calculated as $2^{(-\Delta\Delta Ct)}$. The HCoV-OC43 RNA level was adjusted with the mouse housekeeping gene GAPDH. The primer of HCoV-OC43 and GAPDHA follows:

OC43-S-Forward: GACACCGGTCCTCCTCCTAT;

OC43-S-Reverse: ACACTTCCCTTCAGTGCCAT;

GDPAH-Forward: TGCTGTCCCTGTATGCCTCTG;

GDPAH-Reverse: TTGATGTCACGCACGATTTCC.

12.2 Mouse pharmacokinetic studies[6]

XY4-C7 was administered to C57BL/6 mice in two different studies at doses of 30 mg/kg, 150 μL, either OP or IP. Following administration, 100 μL blood samples were taken at 10 min, 20 min, 30 min, 1 h, 2 h, 4 h, 8 h, 24 h, and 48 h (n = 3 per time point; each mouse was used for three time points, making a total of 9 mice utilized for either OP or IP, making a total of 18 mice.). After drug

administration, 100 μL of blood were collected into 1.5 mL Eppendorf tubes containing 30 μL disodium ED (0.5 M, pH 8.0) and kept on ice until plasma collection (< 30 min), followed by centrifugation at 4000 rpm/min for 10 min at 4 ºC. The supernatants were collected and stored at -80 ºC for future analysis. 50 μL of serum samples were mixed with 15 μL of glacial acetic acid and 135 μL of acetonitrile. The samples were centrifuged at 10,000 rpm and 4 ºC for 15 min after resting on ice for 15 min. Vials containing clarified supernatants were used for the LC/MS/MS analysis. PK parameters were obtained using PKSolver.

13. References

(1) Sang, P.; Shi, Y.; Higbee, P.; Wang, M.; Abdulkadir, S.; Lu, J.; Daughdrill, G.; Chen, J.; Cai, J. Rational design and synthesis of right-handed d-sulfono-γ-AApeptide helical foldamers as potent inhibitors of protein–protein interactions. *J. Org. Chem.* **2020**, *85*, 10552-10560.

(2) Cristóbal-Lecina, E.; Pulido, D.; Martin-Malpartida, P.; Macias, M. J.; Albericio, F.; Royo, M. Synthesis of Stable Cholesteryl–Polyethylene Glycol–Peptide Conjugates with Non-Disperse Polyethylene Glycol Lengths. *ACS omega* **2020**, *5*, 5508-5519.

(3) Ingallinella, P.; Bianchi, E.; Ladwa, N. A.; Wang, Y.-J.; Hrin, R.; Veneziano, M.; Bonelli, F.; Ketas, T. J.; Moore, J. P.; Miller, M. D.; et al. Addition of a cholesterol group to an HIV-1 peptide fusion inhibitor dramatically increases its antiviral potency. *Proc. Natl. Acad. Sci. U.S.A.* **2009**, *106*, 5801-5806.

(4) Abdulkadir, S.; Li, C.; Jiang, W.; Zhao, X.; Sang, P.; Wei, L.; Hu, Y.; Li, Q.; Cai, J. Modulating Angiogenesis by Proteomimetics of Vascular Endothelial Growth Factor. *J. Am. Chem. Soc.* **2022**, *144*, 270-281.

(5) Tian, Y.; Zou, H.; An, P.; Zhou, Z.; Shen, W.; Lin, Q. Design of stapled oxyntomodulin analogs containing functionalized biphenyl cross-linkers. *Tetrahedron* **2019**, *75*, 286-295.

(6) Xue, S.; Wang, X.; Wang, L.; Xu, W.; Xia, S.; Sun, L.; Wang, S.; Shen, N.; Yang, Z.; Huang, B.; et al. A novel cyclic gamma-AApeptide-based long-acting pan-coronavirus fusion inhibitor with potential oral bioavailability by targeting two sites in spike protein. *Cell Discov.* **2022**, *8*, 88.

(7) Xia, S.; Zhu, Y.; Liu, M.; Lan, Q.; Xu, W.; Wu, Y.; Ying, T.; Liu, S.; Shi, Z.; Jiang, S.; et al. Fusion mechanism of 2019-nCoV and fusion inhibitors targeting HR1 domain in spike protein. *Cell. Mol. Immunol.* **2020**, *17*, 765-767.

(8) Sang, P.; Zhou, Z.; Shi, Y.; Lee, C.; Amso, Z.; Huang, D.; Odom, T.; Nguyen-Tran, V. T.; Shen, W.; Cai, J. The activity of sulfono-γ-AApeptide helical foldamers that mimic GLP-1. *Sci. Adv.* **2020**, *6*, eaaz4988.

(9) Xia, S.; Yan, L.; Xu, W.; Agrawal, A. S.; Algaissi, A.; Tseng, C.-T. K.; Wang, Q.; Du, L.; Tan, W.; Wilson, I. A.; et al. A pan-coronavirus fusion inhibitor targeting the HR1 domain of human coronavirus spike. *Sci. Adv.* **2019**, *5*, eaav4580.

(10) Xia, S.; Liu, M.; Wang, C.; Xu, W.; Lan, Q.; Feng, S.; Qi, F.; Bao, L.; Du, L.; Liu, S.; et al. Inhibition of SARS-CoV-2 (previously 2019-nCoV) infection by a highly potent pan-coronavirus fusion inhibitor targeting its spike protein that harbors a high capacity to mediate membrane fusion. *Cell Res.* **2020**, *30*, 343-355.

14. ^{1}H and ^{13}C NMR spectra of sulfonyl-γ-AA peptide building blocks

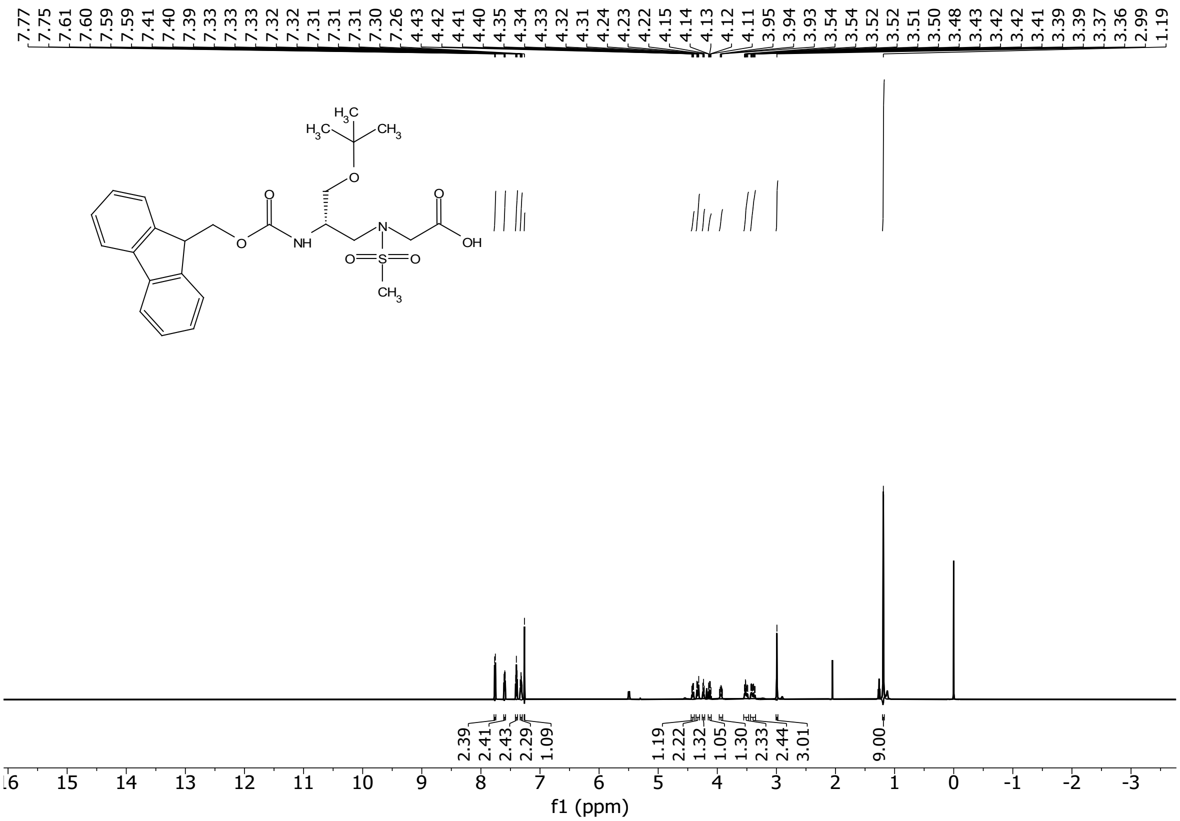

^{1}H NMR of BB7

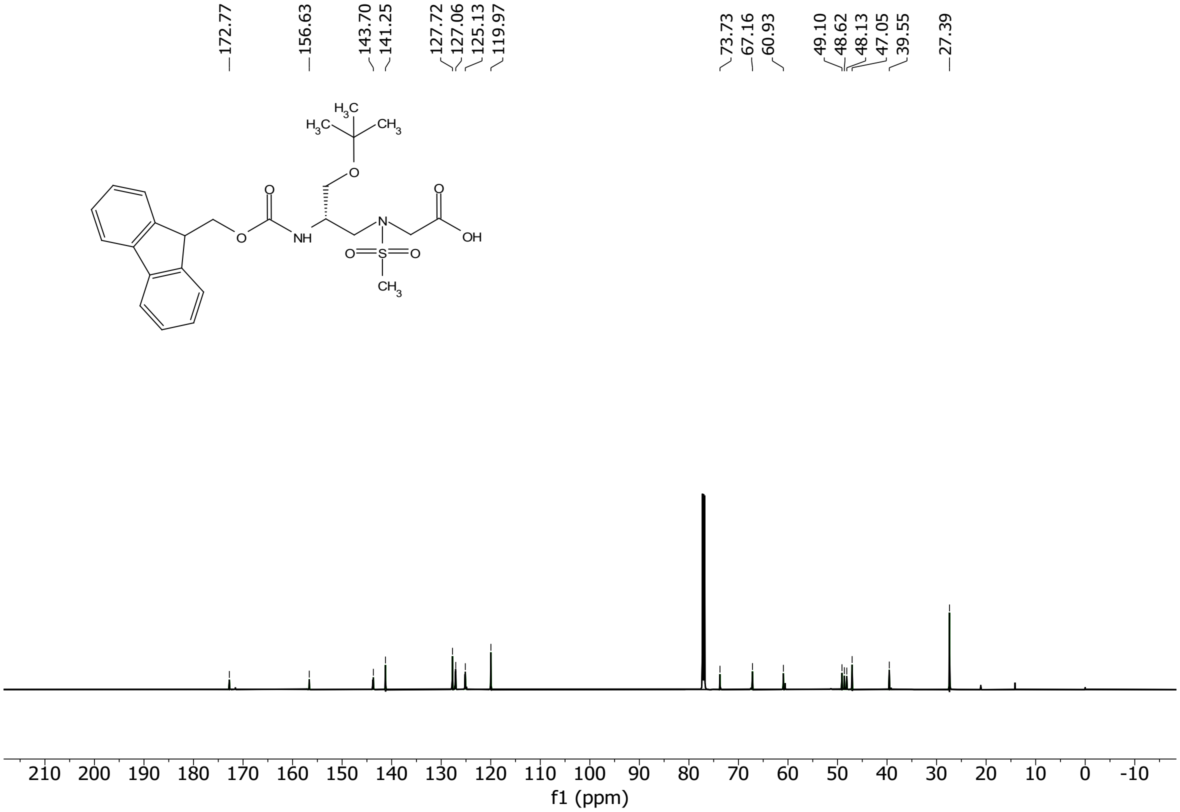

^{13}C NMR of BB7

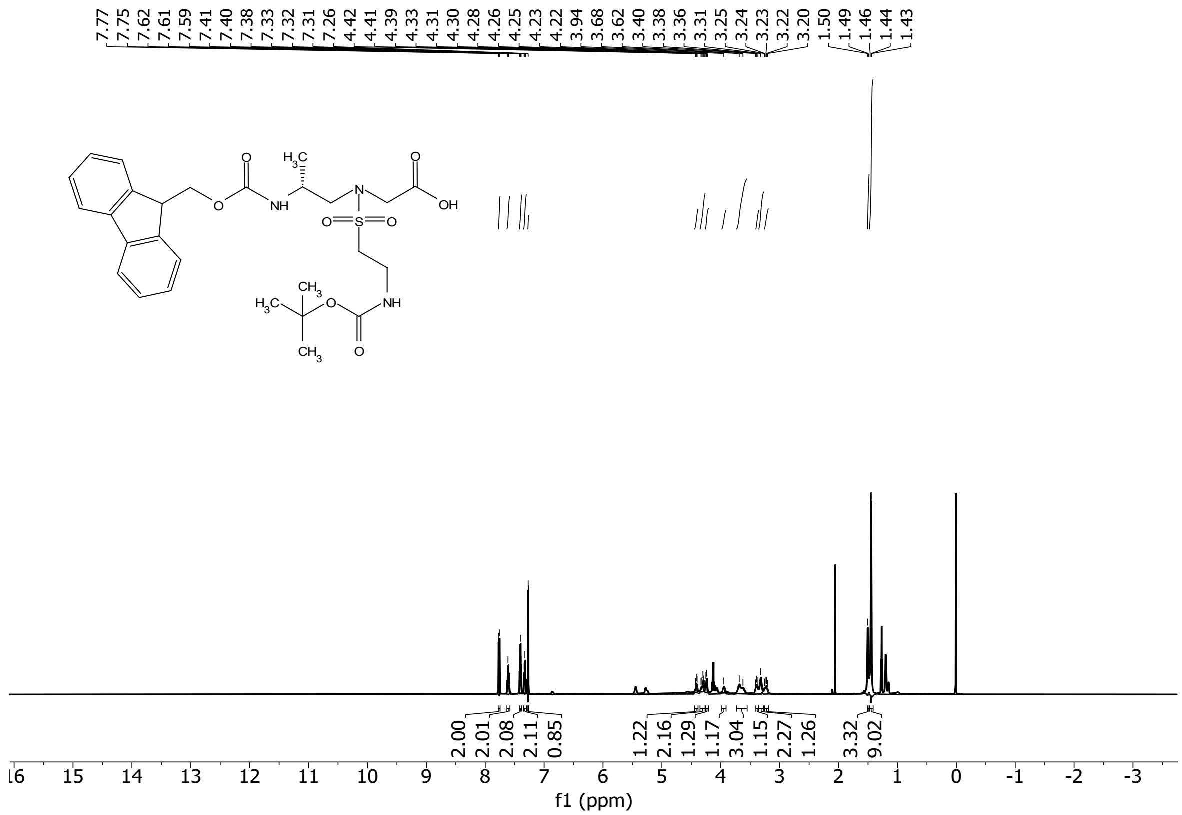

^{1}H NMR of BB8

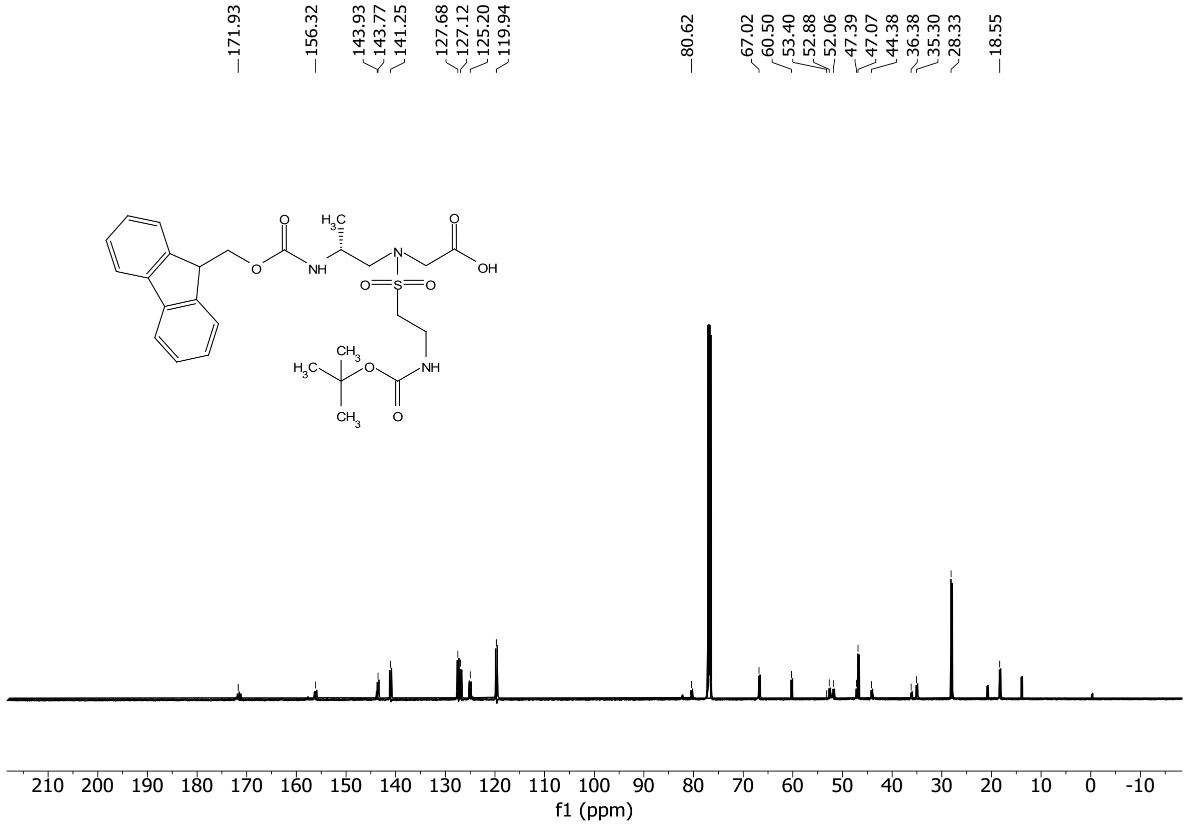

^{13}C NMR of BB8

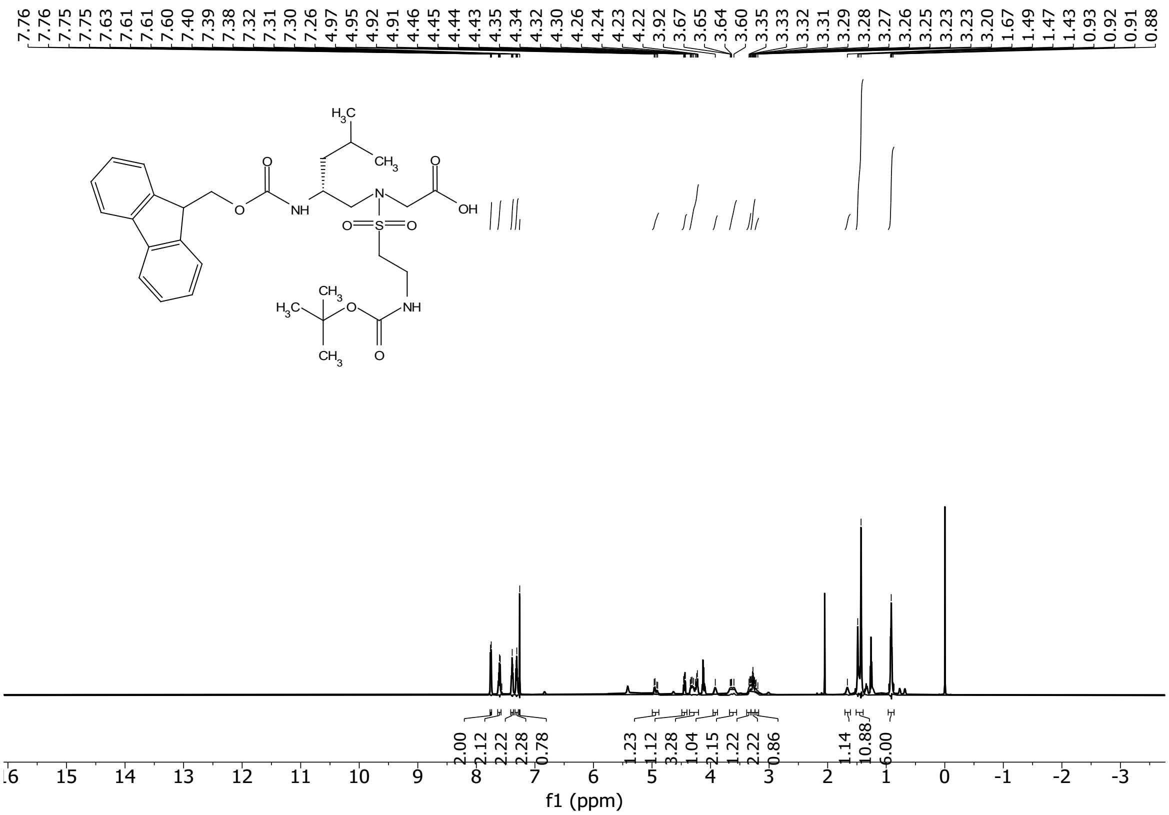

^{1}H NMR of BB10

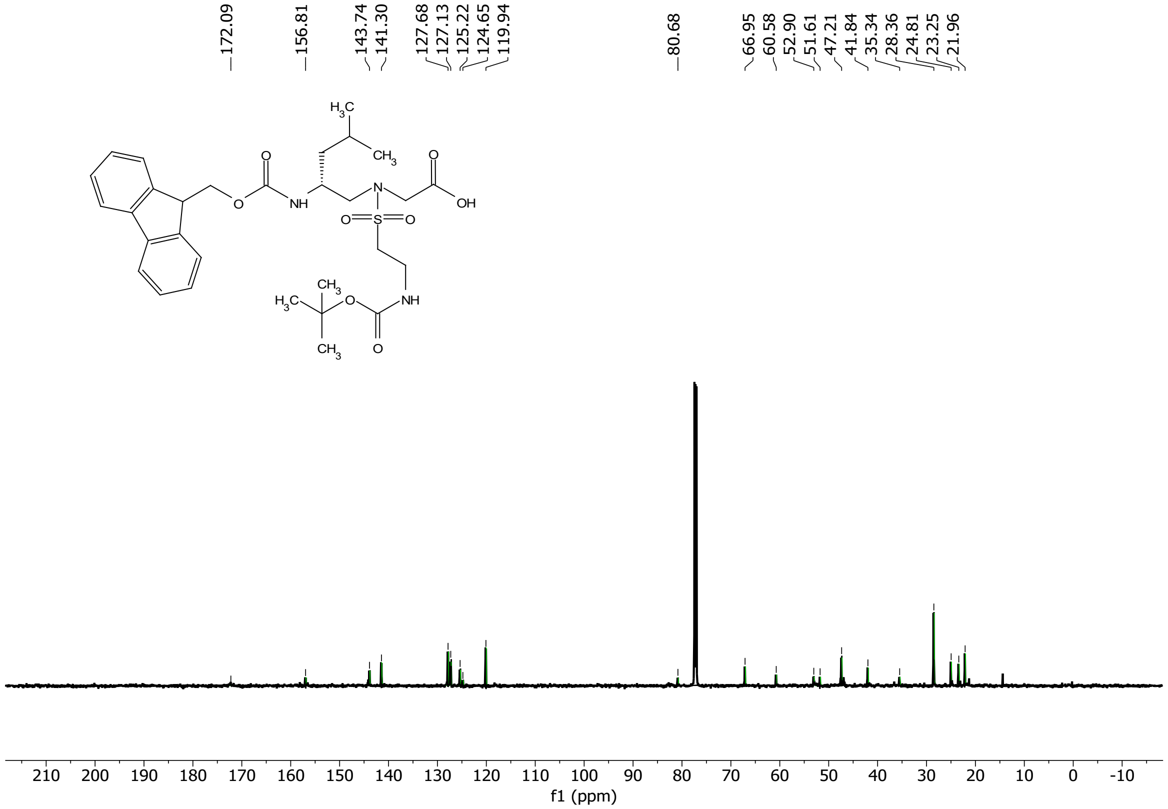

^{13}C NMR of BB10
